# Persistence Length of PEGMA Bottle Brushes Determined by Pyrene Excimer Fluorescence

**DOI:** 10.3390/polym15193958

**Published:** 2023-09-30

**Authors:** Janine L. Thoma, Hunter Little, Jean Duhamel, Lei Zhang, Kam Tong Leung

**Affiliations:** 1 Institute for Polymer Research, Waterloo Institute for Nanotechnology, Department of Chemistry, University of Waterloo, 200 University Avenue West, Waterloo, ON N2L 3G1, Canada; janine.thoma@empa.ch (J.L.T.); htlittle@uwaterloo.ca (H.L.); 2 Waterloo Institute for Nanotechnology, Department of Chemistry, University of Waterloo, 200 University Avenue West, Waterloo, ON N2L 3G1, Canada; l38zhang@uwaterloo.ca (L.Z.); tong@uwaterloo.ca (K.T.L.)

**Keywords:** pyrene excimer fluorescence, persistence length, fluorescence blob model, Kratky–Porod equation

## Abstract

Seven pyrene-labeled poly(oligo(ethylene glycol) methyl ether methacrylate)s (PyEG_5_-PEG_n_MAs) were prepared with *n* = 0, 3, 4, 5, 7, 9, and 19 ethylene glycol units by copolymerizing a small amount of penta(ethylene glycol) 1-pyrenemethyl ether methacrylate with an EG_n_MA monomer. The conformation of the PyEG_5_-PEG_n_MA polymers evolved from a random coil for PyEG_5_-PEG_0_MA or poly(methyl methacrylate) to a polymeric bottle brush (PBB) architecture with increasing side chain length. The fluorescence decays of the PyEG_5_-PEG_n_MA samples were fitted according to the fluorescence blob model (FBM) whose parameters were used, in combination with the Kratky–Porod equation, to calculate the persistence length of these polymers. The persistence lengths obtained from the PEF experiments were found to increase with the square of the number (*N*_S_) of non-hydrogen atoms in the side chain as expected theoretically. The persistence lengths found with the PyEG_5_-PEG_n_MA samples in DMF also matched those found earlier for another series of PEG_n_MA samples labeled with 1-pyrenebutanol. The good agreement found between the persistence lengths obtained with the PEG_n_MA samples labeled with two different pyrene derivatives illustrates the robustness of the method and its applicability for measuring the unknown persistence length of polydisperse polymer samples.

## 1. Introduction

The persistence length (*l*_p_) is a core parameter in polymer science. In lay terms, *l*_p_ reflects how easily a linear chain can bend, with *l*_p_ decreasing with increasing chain flexibility. For instance, *l*_p_ increases from 0.48 nm for flexible poly(ethylene oxide) [1] to 1.8 nm for more rigid bisphenol A polycarbonate [2]. Because stiffer polymers have a larger modulus (*G*), theoretical work has aimed to relate *l*_p_ to *G* to use *l*_p_ for a given polymer as a predictor of the viscoelastic properties expected for its solution [3,4,5,6,7]. Computational methods yield *l*_p_ by measuring the distance over which a vector tangent to the main chain loses its orientation as it is moved along the chain with respect to the tangent vector obtained at a reference point [8]. Experimentally, *l*_p_ is measured by building conformational plots from measurements of the radius of gyration (*R*_G_) [9,10] or the intrinsic viscosity ([*η*]) [11], obtained by scattering or viscosity experiments, respectively, as a function of the molecular weight of polymer samples prepared with a narrow molecular weight distribution (MWD). For many polymers, that cannot be produced with a narrow MWD, gel permeation chromatography (GPC) instruments equipped with a combination of differential refractive index, static light scattering, and viscosity detectors can be employed to generate conformation plots by taking advantage of GPC’s ability to yield *R*_G_ and [*η*] as a function of polymer molecular weight [12,13].

Despite its importance in polymer science, *l*_p_ remains unknown for most polymers because many polymers cannot be obtained with a narrow MWD and require GPC analysis for *l*_p_ determination. Unfortunately, GPC instruments are typically operated in a given solvent, which is not always suitable for all polymer types. Poorly soluble polymers induce interactions between the polymers and the packing material of the GPC column that result in distorted GPC traces, preventing the determination of *l*_p_ by GPC analysis. Consequently, alternative experimental methods are required to determine *l*_p_ for polydisperse polymer samples in solvents where they can be fully dissolved.

The interest in scattering or viscosity techniques for determining *l*_p_ resides in their ability to use *R*_G_ or [*η*] to probe the local density generated inside the macromolecular volume defined by the polymer under study. Since a more flexible polymer can pack more structural units (SU) inside the same macromolecular volume occupied by a stiffer polymer, the polymer coil generated by the stiffer polymer is less dense than the polymer coil generated by the more flexible polymer, thus enabling the determination of *l*_p_ from conformation plots established with *R*_G_ or [*η*]. This discussion suggests that in theory, any technique capable of probing the local density of a polymer coil in solution should be able to yield *l*_p_ for that polymer.

One such technique was recently presented using a methodology based on pyrene excimer formation (PEF) between an excited and a ground-state pyrenyl label covalently attached to a macromolecule. Since PEF is a chemical reaction, its efficiency depends on the local concentration ([*Py*]_loc_) of ground-state pyrenes found within the macromolecular volume [14]. This dependency was recently established by demonstrating that the average rate constant (<*k*>) for PEF between pyrenyl labels attached to a macromolecule is directly proportional to [*Py*]_loc_ [15]. Since the experimentalist knows where the pyrenyl labels are attached on the pyrene-labeled macromolecule (PyLM), [*Py*]_loc_ reflects the local density of a PyLM, thus enabling the application of PEF to determine *l*_p_. The PEF-based methodology that was developed to determine *l*_p_ uses polymers that were randomly labeled with pyrene and whose fluorescence decays were fitted according to the fluorescence *blob* model (FBM) [16]. Within the framework of the FBM, an excited pyrenyl label only probes a subvolume, also referred to as a *blob*, of the much larger polymer coil. The *blob* is then used as a unit volume to compartmentalize the polymer coil into a cluster of *blobs* among which the pyrenyl labels distribute themselves randomly with a Poisson distribution. As for any *blob*-based method, the use of *blobs* shifts the study from the entire polymer to that of a *blob*, eliminating problems associated with polydisperse samples, that often plague scattering studies [17,18] since a large or small polymer can be described by many or few identical *blobs* [19]. The FBM analysis of the fluorescence decays yields the average number <*n*> of pyrenyl groups per *blob*, which is used to determine the number *N*_blob_ of SU per *blob*. Since a flexible polymer can pack more SU inside a *blob* than a stiffer polymer, *N*_blob_ is larger for a flexible polymer than for a stiffer polymer and thus responds to *l*_p_.

With this insight, the dependency of *N*_blob_ on the flexibility, and thus *l*_p_, of a polymer was recently taken advantage of to determine *l*_p_ for a series of poly(oligo(ethylene glycol) methyl ether methacrylate)s, that were labeled with a 1-pyrenebutyl derivative and are referred to as PyBut-PEG_n_MA [20]. However, only *l*_p_ values that are smaller than the dimension of a *blob* probed by an excited pyrenyl label, can be measured with sufficient accuracy. Since the polymer backbone is much less mobile than the pyrenyl label, the linker connecting the pyrene moiety to the polymer backbone defines the size of the *blob*, which can be viewed as a sphere with an equivalent diameter of 3.0 nm in the case of the 1-pyrenebutyl derivative [20]. This meant that *l*_p_ no greater than 2.0 nm could be measured with the PyBut-PEG_n_MA samples [20]. While a range of *l*_p_ values up to 2.0 nm covers a sizeable group of polymers that can be as flexible as PEO with an *l*_p_ of 0.48 nm [1], to bisphenol A polycarbonate with an *l*_p_ of 1.8 nm [2], the ability to determine an *l*_p_ larger than 2.0 nm would enable the study of stiffer backbones. With this in mind, this report describes how labeling PEG_n_MA with a 1-pyrenemethoxy-penta(ethylene glycol) derivative to yield PyEG_5_-PEG_n_MA samples enlarged the diameter of a *blob* from 3.0 nm for the PyBut-PEG_n_MA samples to 5.4 nm in *N*,*N*-dimethylformamide, thus enabling the measurement of *l*_p_ values of up to 4.0 nm. The increase in *l*_p_ values recovered with the PyEG_5_-PEG_n_MA samples from 2.0 to 4.0 nm represents a significant improvement in the range of *l*_p_ values that can be determined from this PEF-based method and it will enable the study of stiffer polymer backbones.

## 2. Materials and Methods

### 2.1. Materials

Copper(II) bromide (Sigma, St Louis, MI, USA, 99%), Celite (Sigma), dichloromethane (DCM, Sigma, ≥99.8%), diethylether (Sigma, ≥99%), *N*,*N*-dimethylformamide (DMF, Sigma, ≥99.8%), dimethyl sulfoxide (DMSO, Sigma, ≥99.9%), 4-(dimethylamino)pyridine (DMAP, Sigma, ≥99%), ethyl acetate (Sigma, ≥99.7%), ethyl-α-bromoisobutyrate (Sigma, 98%), tetra(ethylene glycol) methyl ether (EG_4_, PurePEG, San Diego, CA, USA, ≥97%), penta(ethylene glycol) (Fisher, Hampton, NJ, USA, ≥95%), penta(ethylene glycol) methyl ether (EG_5_, PurePEG, ≥95%), hepta(ethylene glycol) methyl ether (EG_7_, TCI, Portland, OR, USA, ≥97%), 1,1,4,7,10,10-hexamethyl-triethylenetetramine (HMTETA, Sigma, ≥97%), methacrylic anhydride (Sigma, 94%), 1-pyrenemethanol (Sigma, 98%), sodium chloride (Sigma), sodium hydride (NaH, Sigma, 60% dispersion in mineral oil), sodium hydroxide (NaOH, Sigma, pellets, ≥97%), sodium sulfate (Sigma, anhydrous, ≥99%), tetrahydrofuran (Sigma, ≥99%), tetrahydrofuran optima (Fisher, ≥99.9%), and triethylamine (Sigma, ≥99.5%) were used as received.

Tri(ethylene glycol) methyl ether methacrylate (EG_3_MA, Sigma, 93%) and two oligo(ethylene glycol) methyl ether methacrylates (EG_9_MA with number average molecular weight (*M*_n_) = 500 g/mol and EG_19_MA with *M*_n_ = 950 g/mol, Sigma) were dissolved in DCM, washed with 2 M NaOH, and dried with sodium sulfate before use. The radical initiator 2,2′-azo-*bis*-isobutyronitrile (AIBN, Sigma, 98%) was recrystallized in ethanol three times. *p*-Toluenesulfonyl chloride (Sigma, ≥98%) was dissolved in diethyl ether and washed with 2 M NaOH. The organic phase was extracted and dried with sodium sulfate. Unless otherwise specified, all other chemicals were purchased from commercially available sources and used as received.

### 2.2. Preparation of Penta(Ethylene Glycol) Mono p-Toluene Sulfonate (Tos_1_-EG_5_OH)

Penta(ethylene glycol) (EG_5_) (2.00 g, 8.39 mmol) was added to a round bottom flask (RBF) equipped with a magnetic stirrer with freshly distilled DCM. Re-crystallized *p*-toluenesulfonyl chloride (1.76 g, 9.23 mmol) and triethyl amine (1.74 mL, 12.6 mmol) were added to the RBF and left to stir overnight. The next day, the reaction mixture was washed three times with a saturated solution of aqueous sodium chloride. The organic layer was extracted and dried with sodium sulfate. Silica gel chromatography was used to purify the singly tosylated EG_5_ (Tos_1_-EG_5_OH) from the doubly tosylated EG_5_ and unmodified EG_5_ using ethyl acetate as the eluent. The Tos_1_-EG_5_OH fraction was dried in vacuo (vacuum oven from VWR, Radnor, PA, USA) and its chemical composition was verified using ^1^H NMR (Figure S1 in SI).

### 2.3. Preparation of 1-Pyrenemethyl Ether Penta(Ethylene Glycol) (PyEG_5_OH)

1-Pyrenemethanol (1.18 g, 5.10 mmol) was added to a RBF with 50 mL of dried and distilled DMF. The solution was stirred and kept under a nitrogen atmosphere. Sodium hydride (NaH) (0.20 g, 5.10 mmol) was added to the RBF and the solution was allowed to stir for 1 h during which time the color of the solution changed from yellow to dark red/purple. Tos_1_-EG_5_OH (1.00 g, 2.55 mmol) was then added. The RBF was placed in an oil bath at 55 °C and left to stir overnight. After the RBF was removed from the oil bath and allowed to cool, 5 mL of Milli-Q water was added to the reaction solution to quench any unreacted NaH. Milli-Q water (50 mL) was then added to the reaction mixture, which was washed with 50 mL of ethyl acetate. The organic phase was collected and dried with sodium sulfate. The crude product was purified by silica gel chromatography using ethyl acetate as the eluent. The chemical composition of the purified PyEG_5_OH product was confirmed using ^1^H NMR (Figure S2 in SI).

### 2.4. Methacrylation of Oligo(Ethylene Glycol) Methyl Ethers (EG_n_OHs) and PyEG_5_OH

The same protocol was used to prepare the methacrylated oligo(ethylene glycol) methyl ethers (EG_n_MAs, where *n* = 4, 5, 7) and PyEG_5_MA. The synthesis of EG_5_MA is described in more detail hereafter.

EG_5_OH (2.00 g, 7.93 mmol) and DMAP (0.0968 g, 0.793 mmol) were added to a RBF with 25 mL of freshly distilled DCM. The RBF was then placed in an ice water bath and the solution was stirred as methacrylic anhydride was added dropwise (1.18 mL, 7.93 mmol). The reaction was left to stir overnight. The reaction mixture was then washed three times with 2 M NaOH. The organic phase was extracted and dried with sodium sulfate. The crude product was purified by silica gel chromatography using ethyl acetate as the eluent. The chemical composition of the purified EG_5_MA macromonomer was characterized by ^1^H NMR (Figure S3 in SI).

### 2.5. Random Copolymerization Using Conventional Radical Polymerization

The pyrene-labeled poly(oligo(ethylene glycol) methyl ether methacrylate)s (PyEG_5_-PEG_n_MA) were prepared by conventional radical polymerization of methyl methacrylate (EG_0_MA), tri(ethylene glycol) methyl ether methacrylate (EG_3_MA), tetra(ethylene glycol) methyl ether methacrylate (EG_4_MA), penta(ethylene glycol) methyl ether methacrylate (EG_5_MA), hepta(ethylene glycol) methyl ether methacrylate (EG_7_MA), and two oligo(ethylene glycol) methyl ether methacrylates (EG_9_MA and EG_19_MA) with PyEG_5_MA. The chemical structure of the PyEG_5_-PEG_n_MA samples is shown in Table 1. The moles of PyEG_5_MA used in the polymerization were varied to obtain different molar percentages of pyrene-labeling, ranging from 1 to 10 mol % of PyEG_5_MA, incorporated into the PyEG_5_-PEG_n_MA samples. The polymerization of PyEG_5_-PEG_0_MA labeled with 2 mol % of PyEG_5_MA is described in more detail hereafter.

PyEG_5_MA (0.02 g, 0.04 mmol) and methyl methacrylate (EG_0_MA, 0.20 g, 2.00 mmol) were dissolved in 6.8 mL of THF such that the overall methacrylate concentration was approximately 0.3 M. The AIBN initiator (2.00 μg, 0.01 μmol) was added to the monomer solution from a stock solution and the mixture was placed in the polymerization tube. The tube was kept on ice before being degassed with nitrogen (Praxair, Danburry, CT, USA, N4.0) for 30 min. After sealing the tube, it was left in an oil bath at 65 °C. The polymerization was terminated after a conversion of 20% or less was reached, as determined by ^1^H NMR analysis, to minimize an eventual composition drift. The polymer was recovered by precipitating 5–6 times the polymer solution in THF into diethyl ether to remove any unreacted monomer. The precipitated product was then dried in a vacuum oven overnight at room temperature.

### 2.6. Random Copolymerization Using Initiators for Continuous Activator Regeneration Atom Transfer Radical Polymerization (ICAR-ATRP)

Three of the PyEG_5_-PEG_19_MA samples were prepared using ICAR-ATRP [21]. The protocol described for free radical copolymerization was applied to prepare PyEG_5_-PEG_19_MA using ethyl-α-bromoisobutyrate, Cu(II)Br/HMTETA, and AIBN as initiator, catalyst/ligand system, and radical source, respectively. An example of the ICAR-ATRP of PyEG_5_-PEG_19_MA is provided in more detail hereafter.

A solution of PyEG_5_MA (0.02 g, 0.04 mmol) and EG_19_MA (1.00 g, 1.05 mmol) in 3.6 mL of THF, where the overall methacrylate concentration equaled 0.3 M, was transferred to the polymerization tube. A stock solution of Cu(II)Br (1.18 mg, 5.28 μmol) and HMTETA (4.3 μL, 15.8 μmol) was prepared in 10 mL of THF from which 10 μL was added to the polymerization tube. Ethyl-α-bromoisobutyrate (10.0 μL, 68.1 μmol) was added to 1 mL of THF from which 7.8 μL was added to the polymerization tube. AIBN (5.00 mg, 0.03 mmol) was added to 10 mL of THF to make a 3.05 mM stock solution. The solution was further diluted to 0.3 mM from which 0.2 mL was added to the polymerization tube, which was placed on ice and degassed for 30 min with nitrogen (Praxair, N4.0). The tube was then sealed and heated to 60 °C in an oil bath for 20 h. Before the polymer was precipitated, the polymer solution in THF was filtered through a silica gel and Celite plug three times to remove copper. The polymer was further purified by 5–6 precipitations into diethyl ether.

### 2.7. Chemical Composition and Molecular Weight Distribution

The chemical composition of the PyEG_5_-PEG_n_MA polymers was confirmed by the analysis of the ^1^H NMR spectra acquired on a Bruker 300 MHz high resolution spectrometer (Bruker, Billerica, MA, USA). A sample ^1^H NMR spectrum of PyEG_5_-PEG_5_MA is provided in Figure S4 in the SI. The molecular weight distribution (MWD) of each PyEG_5_-PEG_n_MA sample was determined by GPC analysis using either THF or DMSO. The pyrene contents (in mol%), *M*_n_, and dispersity (*Ð*) of each sample are listed in Table 2.

### 2.8. Pyrene Content of PyEG_5_-PEG_n_MA Samples

The pyrene content expressed as the molar fraction (*x*) of the PyEG_5_MA monomer incorporated in the copolymers, equivalent to the molar fraction of structural units bearing a pyrenyl labels, was calculated with Equation (1).

(1) $$x=\frac{M}{\lambda_{Py}^{-1}+M-M_{Py}}$$

In Equation (1), *λ*_Py_, *M*, and *M*_Py_ are the pyrene content of the polymer expressed in mol of pyrene per gram of polymer and the molar mass of the EG_n_MA and PyEG_5_MA monomers, respectively. λ_Py_ was determined as follows. A polymer solution was prepared in THF with a known mass concentration (*m*) of a PyEG_5_-PEG_n_MA sample. The pyrene content of the polymer (λ_Py_) was calculated from the ratio *Abs*/(*m* × *ε*), where *Abs* is the absorption at 344 nm of the PyEG_5_-PEG_n_MA solution in THF and *ε* is the molar absorption coefficient of 1-pyrenemethanol in THF (ε(344 nm) = 42,700 M^−1^·cm^−1^) [22].

### 2.9. Gel Permeation Chromatography (GPC)

Absolute molecular weights were obtained for PyEG_5_-PEG_0_MA, PyEG_5_-PEG_3_MA, and PyEG_5_-PEG_5_MA by injecting 1 mg/mL solutions of the samples dissolved in THF into a Viscotek GPC (Viscotek, Houston, TX, USA) equipped with a differential refractive index, static light scattering (low and right angle), and UV–Vis absorption detector and three 300 × 8 mm^2^ PolyAnalytik Superes linear mixed-bed columns (PolyAnalytik, London, ON, Canada). A flow rate of 1 mL/min of THF at 35 °C was used. The system was calibrated with a 1 mg/mL THF solution of a polystyrene (PS) standard with *M* = 90 × 10^3^ g·mol^−1^ and *Ð* = 1.04.

However, PyEG_5_-PEG_7_MA, PyEG_5_-PEG_9_MA, and PyEG_5_-PEG_19_MA were found to interact with the column set of the GPC instrument in THF resulting in distorted GPC traces. As a result, the absolute molecular weights of these samples were obtained by injecting 2 mg/mL polymer solutions in DMSO into a TOSOH EcoSEC High Temperature GPC instrument equipped with a triple detection system and two 300 × 7.8 mm^2^ TOSOH TSKgel Alpha-M 13 μm columns (Tosoh, Tokyo, Japan). This detection system included an in-line differential refractometer, a Wyatt Dawn Heleos8 MALLS detector (wavelength, λ = 660 nm) (Wyatt, Santa Barbara, CA, USA), and a viscometer. A flow rate of 0.6 mL/min of DMSO at 60 °C was used. The system was calibrated with a 1 mg/mL solution of pullulan standard in DMSO with *M*_w_ = 47.1 × 10^3^ g·mol^−1^ and *Ð* = 1.07.

The specific refractive index increment (*d*n*/d*c) of each polymer in THF and DMSO was calculated using the differential refractometers of the GPC instruments. Sample GPC traces can be found in Figure S5 in SI.

### 2.10. Atomic Force Microscopy (AFM)

AFM images were obtained with a Digital Instruments Dimension 3100 AFM (Digital Instruments, Santa Barbara, CA, USA) at room temperature using a silicon cantilever in the tapping mode. The samples were prepared by spin coating a few drops of a dilute solution of polymer dissolved in tetrahydrofuran (THF) (10 mg/L) onto a freshly cleaved mica surface at 2000 rpm.

### 2.11. UV–Vis Spectroscopy

A Varian Cary 100 Bio spectrophotometer (Varian, Palo Alto, CA, USA) was used to acquire the absorption spectra of the polymer solutions.

### 2.12. Steady-State Fluorescence (SSF) Measurements

All fluorescence spectra were acquired on a Horiba QM-400 spectrofluorometer equipped with a Xenon arc lamp (HORIBA Canada, Burlington, ON, Canada). The SSF spectra were acquired for polymer solutions in aerated DMSO with a 2.5 × 10^−6^ M pyrene concentration equivalent to an absorbance of ~0.1 at 344 nm. The solutions were excited at 344 nm and scanned from 350 to 600 nm using 1 nm slit widths for both the excitation and emission monochromator. Dividing the florescence intensity of the excimer (*I*_E_) by the fluorescence intensity of the monomer (*I*_M_), calculated by integrating the area underneath the spectrum from 500 to 510 nm and from 375 to 381 nm, respectively, yielded the *I*_E_/*I*_M_ ratio, which was used to quantify the efficiency of pyrene excimer formation (PEF).

### 2.13. Time Resolved Fluorescence (TRF) Measurements

All fluorescence decays were obtained with an IBH time-resolved fluorometer (IBH, Glasgow, SCT, UK). The solutions were excited at 344 nm and the monomer and excimer fluorescence decays were acquired with 20,000 counts at the decay maximum over 1024 channels at 375 and 510 nm using cut-off filters at 370 and 495 nm, respectively. A time-per-channel of either 1.02 ns/ch or 2.04 ns/ch was employed for the decay acquisition. A Ludox solution was used for the instrument response function (IRF), which was obtained by setting the emission monochromator at 344 nm. The IRF was convoluted with the FBM equations shown as Equations (S1) and (S2) in SI and the convolution result was compared to the experimental decay.

### 2.14. The Fluorescence Blob Model (FBM) Analysis

The FBM compartmentalizes a polymer into equally sized *blobs*, where the volume of a *blob* is the volume probed by a pyrenyl label, while it remains excited [14,16]. The four pyrene species *Py*_diff_*, *Py*_k2_*, *Py*_agg_*, and *Py*_free_* are considered to represent PEF, which occurs via a dynamic and static pathway. Dynamic PEF takes place sequentially. *Py*_diff_* represents an excited pyrenyl group, whose diffusion in solution is controlled by the polymer backbone and side chain dynamics. *Py*_diff_* diffuses inside a *blob* populated by other ground-state pyrenes until *Py*_diff_* becomes close enough to a ground-state pyrene molecule for *Py*_diff_* to turn into *Py*_k2_*. The diffusive motions of two pyrenyl groups inside a *blob* are described by the rate constant *k*_blob_. Rapid rearrangement of *Py*_k2_* and the nearby ground-state pyrene with the large rate constant *k*_2_ (*k*_2_~10 × *k*_blob_) results in the formation of an excimer made of two pyrenyl labels, that are well (*E*0*), or poorly (D*) stacked and emit with their natural lifetimes *τ*_E0_ and *τ*_D_, respectively. Static PEF occurs through direct excitation of a pyrene aggregate resulting in the instantaneous formation of the *E*0* or D* species. The species *Py*_agg_* combines the two pyrenyl species *E*0* and D* formed instantaneously from the direct excitation of a pyrene aggregate. Finally, those excited pyrenes, that are isolated along the polymer backbone, do not form excimers, and emit with their natural lifetime *τ*_M_, and are referred to as *Py*_free_*. During the decay analysis, the decays are fit twice, initially with a floating *k*_2_ using the program *globmis90lbg* for all samples of a same PyEG_5_-PEG_n_MA series prepared with different pyrene contents. All *k*_2_ values obtained for a same polymer series are then averaged and the averaged *k*_2_ value is then fixed in a second analysis with the program *globmis90obg*. The parameters retrieved from the FBM analysis with a fixed *k*_2_ have much lower error bars. The molar fractions *f*_Mdiff_, *f*_Mk2_, *f*_Mfree_, where the index *M* indicates that they were derived from the monomer decays, and *f*_Ek2_, *f*_EdiffE0_, *f*_EE0_, *f*_EdiffD_, and *f*_ED_, where the index *E* indicates that they were derived from the excimer decays, were combined to yield the molar fractions *f*_diff_ (=*f*_diffE0_ + *f*_diffD_), *f*_k2_, *f*_agg_ (=*f*_E0_ + *f*_D_), and *f*_free_ for the pyrene species *Py*_diff_*, *Py*_k2_*, *Py*_agg_*, and *Py*_free_*, respectively. The average number (<*n*>) of ground state pyrene molecules inside a *blob* and the rate constant (*k*_blob_) describing the diffusive encounters of two structural units bearing a pyrenyl label inside a *blob* were also obtained from the FBM analysis. The number (*N*_blob_) of structural units encompassed within a *blob* was calculated using *f*_Mfree_, <*n*>, and *x* according to Equation (2).

(2) $$N_{\mathrm{blob}}=\frac{(1-f_{M_{free}})\times \langle n\rangle }{x}$$

Each pair of monomer and excimer fluorescence decays acquired for a given PyEG_5_-PEG_n_MA sample was fit globally with Equations (S1) and (S2) in SI according to the FBM. The functions described by Equations (S1) and (S2) were convoluted with the IRF and the convolution product was compared to the experimental decays for optimization of the parameters with the Marquardt–Levenberg algorithm [23]. A fit was deemed acceptable when the χ^2^ was lower than 1.3 and when both the residuals and autocorrelation of the residuals were randomly distributed around zero.

### 2.15. Flow Chart Depicting the Methodology Applied for Determining the Persistence Length

**Figure 1:**
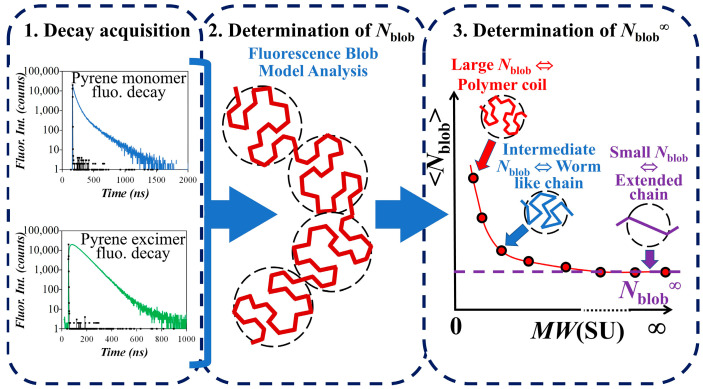
Depiction of the experimental process applied to determine <*N*_blob_> and *N*_blob_^∞^ used to retrieve *l*_p_.

**Figure 2:**
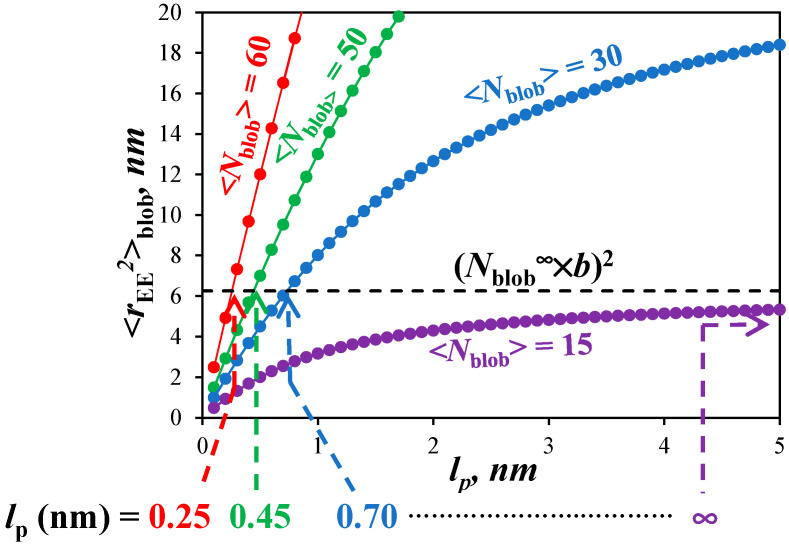
Geometric construction depicting the mathematical procedure applied to retrieve *l*_p_ from <*N*_blob_> and *N*_blob_^∞^ determined in Figure 1.

The strategy applied to determine the persistence length by PEF is depicted in Figure 1 and Figure 2. Figure 1 represents the experimental process to determine <*N*_blob_> for each PyEG_5_-PEG_n_MA series and *N*_blob_^∞^, which is the <*N*_blob_> value obtained for a hypothetical PyEG_5_-PEG_n_MA sample having an infinitely long side chain (*n* → ∞). Figure 2 is a geometrical construction describing the mathematical process applied to determine *l*_p_ from <*N*_blob_> according to the Kratky–Porod equation [24]. In Figure 1, the fluorescence decays of the pyrene monomer and excimer shown in the left panel are acquired and fitted globally according to the FBM illustrated in the middle panel to determine *N*_blob_ for different PyEG_5_-PEG_n_MA samples of a same series with *n* = 0, 3, 4, 5, 7, 9, and 19. The *N*_blob_ values obtained for several pyrene contents of a same PyEG_5_-PEG_n_MA series are averaged to obtain <*N*_blob_>. These <*N*_blob_> values are plotted as a function of the molecular weight of a structural unit (*MW*(SU)) in the right panel of Figure 1. For small *MW*(SU), *N*_blob_ is large indicating a coiled conformation reflecting a small *l*_p_. As *MW*(SU) increases, *N*_blob_ decreases as the chain conformation changes from a coiled to a worm-like conformation. For very large *MW*(SU), the polymer chain achieves an extended conformation on the length-scale of the *blob*, *N*_blob_ does not change any more with increasing *MW*(SU), and its value corresponds to that expected for an extended polymer with infinitely long side chains (*N*_blob_^∞^).

Once <*N*_blob_> and *N*_blob_^∞^ are determined, their values are introduced into Equation (3), which is a modified version of the Kratky–Porod equation (KPE) for worm-like chains to account for the fact that it is applied to the chain segment inside a *blob* instead of the entire chain. The left part of the KPE in Equation (3) represents the square of the end-to-end distance (<*r*_EE_^2^>_blob_) of the chain fragment encompassed inside a *blob*. Since the pyrene moiety is connected to the polymethacrylate backbone with the same linker for all PyEG_5_-PEG_n_MA constructs, all PyEG_5_-PEG_n_MA samples share a same *blob* regardless of side chain length. According to this reasoning, <*r*_EE_^2^>_blob_ takes the same value for all PyEG_5_-PEG_n_MA samples including those samples that have an infinitely long side chain for which the polymethacrylate backbone is fully extended. The fully extended polymethacrylate backbone corresponds to a hypothetical PyEG_5_-PEG_n_MA sample, for which *n* and *MW*(SU) take infinitely large values and *N*_blob_ tends to *N*_blob_^∞^ as shown in the right panel of Figure 1. For a fully extended chain segment inside a *blob*, <*r*_EE_^2^>_blob_ equals the product (*N*_blob_^∞^ × *b*)^2^, where *b* is the length of a methacrylate structural unit equal to 0.25 nm [25,26]. Since all PyEG_5_-PEG_n_MA share the same *blob* with <*r*_EE_^2^>_blob_ = (*N*_blob_^∞^ × *b*)^2^, the left-hand side of the KPE is known and the function on the right-hand side of the equation can be solved for *l*_p_ as represented in Figure 2. The <*N*_blob_> value obtained for each PyEG_5_-PEG_n_MA series is entered into the right-hand side of the KPE, which is plotted as a function of *l*_p_ in Figure 2. The abscissa of the intercept between the horizontal dashed line representing <*r*_EE_^2^> = (*N*_blob_^∞^ × *b*)^2^ and the line corresponding to the right-hand side of the KPE yields *l*_p_ for the PyEG_5_-PEG_n_MA series under consideration.

(3) $$<r_{EE}^{2}{>}_{blob}\;=\;{\left(N_{blob}^{\infty }\times b\right)}^{2}\;=\;2l_{p}(b\times <N_{blob}>)\;-\;2l_{p}^{2}\left[1\;-\;\exp\left(-\frac{b\times <N_{blob}>}{l_{p}}\right)\right]$$

## 3. Results and Discussion

A series of pyrene-labeled poly(oligo(ethylene glycol) methyl ether methacrylate)s (PyEG_5_-PEG_n_MA with *n* = 0, 3, 4, 5, 7, 9, and 19) were synthesized using a grafting through technique by mainly free radical copolymerization of the same penta(ethylene glycol) 1-pyrenemethyl ether methacrylate (PyEG_5_MA) and different oligo(ethylene glycol) methyl ether methacrylate (EG_n_MA) macromonomers. Their chemical structure, the number (*N*_S_) of atoms in each side chain, and the molecular weight of the structural unit (*MW*(SU)) were presented in Table 1. The number average molecular weight (*M*_n_) and dispersity (*Ð*) of all PyEG_5_-PEG_n_MA samples were determined by gel permeation chromatography and are listed in Table 2. Variations in *M*_n_ and *Ð* were observed from sample-to-sample in Table 2 due to the relative purity and reactivity of the different monomers. Nevertheless, the *M*_n_ values were sufficiently large to ensure that all polymer samples were constituted of many *blobs*, which enabled the analysis of the fluorescence decays with the FBM, that could handle these samples, whose *Ð* values greater than 1.0 indicate that they are polydisperse. The design of the PyEG_5_-PEG_n_MA constructs was carefully considered. It was established in an earlier study with pyrene-labeled poly(*n*-butyl methacrylate)s, that the motion of the pyrenyl group became uncorrelated from the motion of the main chain, when a 1-pyrenemethoxy derivative was connected to the main polymethacrylate backbone by a linker made of two or more ethylene glycol units [27]. The use of a penta(ethylene glycol) linker for the PyEG_5_-PEG_n_MA samples thus ensured that an excited pyrenyl label would probe a well-defined sub-volume (*V*_blob_) of the PBBs, referred to as a *blob* within the FBM framework, that would be unaffected by any main chain motion. In turn, this condition implied that each PBB was being probed over the same length scale defined by the same *V*_blob_ for all PyEG_5_-PEG_n_MA constructs considered in this study.

**Figure 3:**
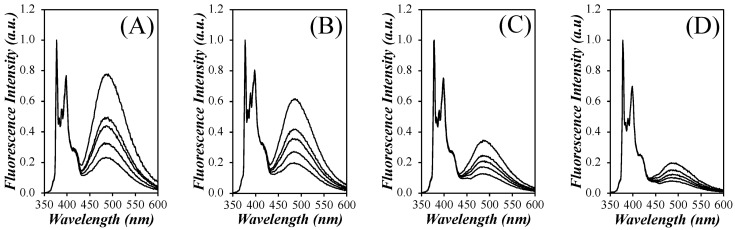
Steady-state fluorescence spectra of the PyEG_5_-PEG_4_MA samples in (**A**) acetonitrile, (**B**) THF, (**C**) DMF, and (**D**) DMSO with pyrene contents ranging from 1.2 to 3.1 mol % shown from bottom to top. [*Py*] = 2.5 × 10^−6^ M; *λ*_ex_ = 344 nm.

The SSF spectra for all pyrene contents of each PyEG_5_-PEG_n_MA series were acquired in acetonitrile, tetrahydrofuran (THF), *N*,*N*-dimethylformamide (DMF), and dimethyl sulfoxide (DMSO) and are presented in Figures S6–S9 in the SI. The spectra for all pyrene contents of the PyEG_5_-PEG_4_MA series in each solvent are shown in Figure 3.

The spectra were normalized to the first peak of the monomer emission, *I*_1_, which is the 0-0 transition of pyrene. They showed the characteristic fluorescence peaks between 375 and 410 nm for the pyrene monomer with the broad and structureless excimer emission centered at 480 nm. It is apparent from Figure 3, that more excimer is produced in acetonitrile than in THF, DMF, and DMSO, with DMSO producing the least amount of excimer. The *I*_E_/*I*_M_ ratio was calculated to quantify the efficiency of pyrene excimer formation (PEF) for the different constructs in different solvents. The *I*_E_/*I*_M_ ratio is proportional to the local concentration of pyrene, [*Py*]_loc_, and the rate constant for PEF through diffusive encounters, *k*_diff_, as indicated by Equation (4).

(4) $$\frac{I_{E}}{I_{M}}\;\sim \;k_{diff}\times {\left[Py\right]}_{loc}$$

**Figure 4:**
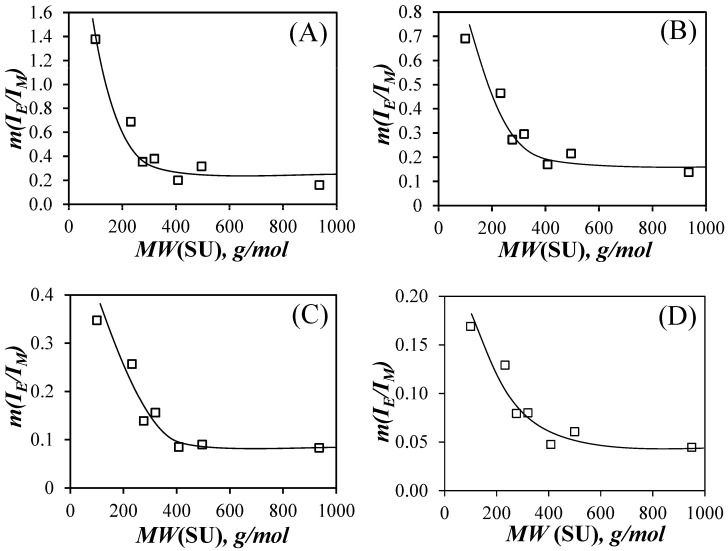
Plot of *m*(*I*_E_/*I*_M_) versus *MW*(SU) for the PyEG_5_-PEG_n_MA PBBs in (**A**) acetonitrile, (**B**) THF, (**C**) DMF, and (**D**) DMSO.

The *I*_E_/*I*_M_ ratios were plotted as a function of pyrene content for each PyEG_5_-PEG_n_MA sample in Figure S10. They yielded straight lines over a wide range of pyrene contents and the slope of these lines (*m*(*I*_E_/*I*_M_)) was plotted as a function of *MW*(SU) in Figure 4. In each solvent, the slope *m*(*I*_E_/*I*_M_) decreased as *MW*(SU) increased for the PyEG_5_-PEG_n_MA samples with *n* equal to 0, 3, 4, and 5, respectively. This decrease was attributed to an extension of the polymer backbone, that resulted from increased crowding of the volume surrounding the main chain with increasing *MW*(SU). Main chain extension reduced the number of encounters between the pyrenyl terminals of the PyEG_5_ side chains, which was associated with a decrease in [*Py*]_loc_ in Equation (3). The decrease in *m*(*I*_E_/*I*_M_) continued until an *MW*(SU) of 408 g/mol for PyEG_5_-PEG_7_MA was reached, after which *m*(*I*_E_/*I*_M_) seemed to plateau for *MW*(SU) values of 500 and 950 g/mol for PyEG_5_-PEG_9_MA and PyEG_5_-PEG_19_MA, respectively. The plateau region observed for *N*_S_ values larger than 400 g/mol indicated that a further increase in side chain length would not result in an increase in main chain extension, probably because the main chain was, or was close to being, fully extended on the length scale probed by an excited pyrene. The *m*(*I*_E_/*I*_M_)-*vs.*-*MW*(SU) trends shown in Figure 4 suggested that the steric hindrance generated by the side chains influence a region inside the polymeric bottlebrush (PBB) volume, that is close to the main chain and where the shorter EG_n_ side chains have the strongest effect. As the side chains become long enough to expand past the local region close to the main chain and into the mostly empty space away from the main chain, their effect on the main chain becomes less important, resulting in the plateau observed for large side chain lengths in the *m*(*I*_E_/*I*_M_)-*vs*-*MW*(SU) plot in Figure 4. Similar saturation effects with increasing side chain length have already been reported for PBBs [20,28].

The *m*(*I*_E_/*I*_M_) slopes in Figure 4 were also found to be larger in acetonitrile, followed by THF, DMF, and DMSO. This trend reflects the influence of the solvent viscosity, *η*. The viscosity of acetonitrile, THF, DMF, and DMSO at 25 °C equals 0.37, 0.46, 0.79, and 1.99 mPa·s, respectively [29]. Since *k*_diff_ is inversely proportional to solvent viscosity [30], acetonitrile with the lowest *η* yielded the largest *k*_diff_ values in Equation (4) and the largest *m*(*I*_E_/*I*_M_) slopes in Figure 4A. Similarly, DMSO being the most viscous solvent yielded the lowest *m*(*I*_E_/*I*_M_) slopes in Figure 4D. THF and DMF with their intermediate *η* values resulted in intermediate *m*(*I*_E_/*I*_M_) slopes. As was pointed out in earlier reports [31,32], solvent viscosity, while important, is not the only parameter affecting *k*_diff_. The probability *p*, of forming an excimer upon an encounter between an excited and a ground-state pyrenyl label, depends also on the solvent, and its value can offset the relationship expected between *k*_diff_ and *η*^−1^ [30]. Consequently, the interpretation of the parameter *m*(*I*_E_/*I*_M_) obtained from the analysis of the steady-state fluorescence spectra offers only a qualitative description of the fluorescence results.

**Figure 5:**
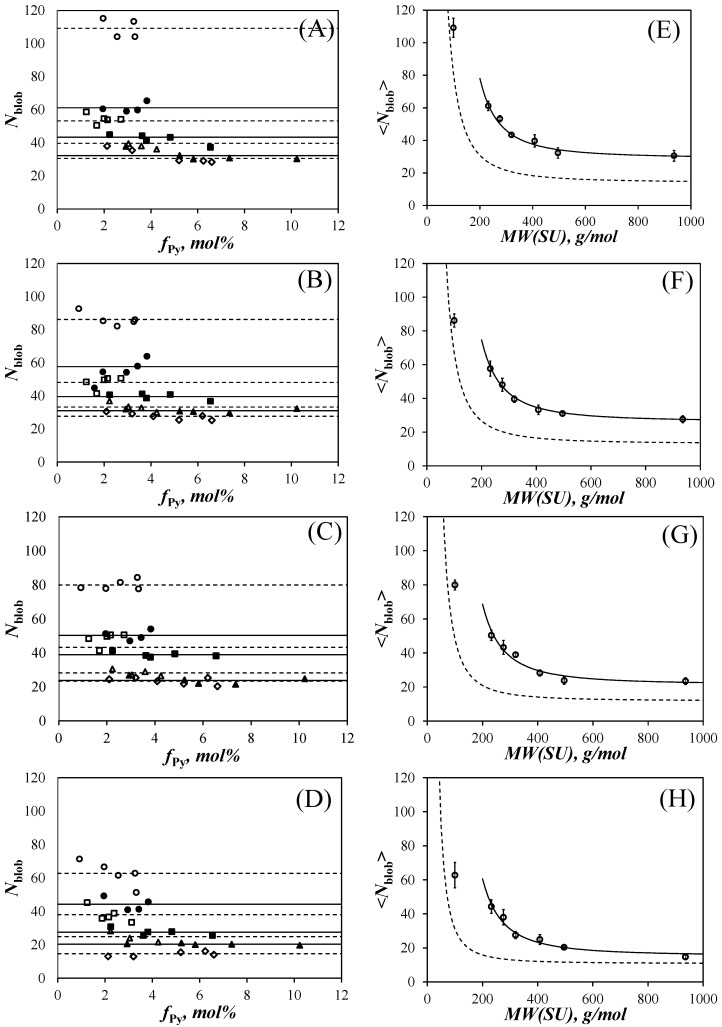
Plots of (**A**–**D**) *N*_blob_ as a function of pyrene content for (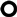) PyEG_5_-PEG_0_MA, (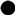) PyEG_5_-PEG_3_MA, (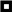) PyEG_5_-PEG_4_MA, (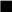) PyEG_5_-PEG_5_MA, (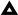) PyEG_5_-PEG_7_MA, (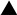) PyEG_5_-PEG_9_MA, and (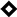) PyEG_5_-PEG_19_MA and (**E**–**H**) <*N*_blob_> as a function of *MW*(SU) in (**A**,**E**) acetonitrile, (**B**,**F**) THF, (**C**,**G**) DMF, and (**D**,**H**) DMSO. Lines: prediction for the <*N*_blob_> values for (solid) the PyEG_5_-PEG_n_MA samples with Equation (8) and (dashed) the PyBut-PEG_n_MA samples.

A more quantitative measure of polymer stiffness, such as the persistence length (*l*_p_), can only be retrieved from PEF measurements through the global analysis of the monomer and excimer decays acquired with the PyEG_5_-PEG_n_MA samples as was performed earlier with the PyBut-PEG_n_MA samples [20]. The determination of *N*_blob_ for the PyEG_5_-PEG_n_MA samples represents the second step in the procedure applied to obtain *l*_p_ as described in Figure 1. The FBM analysis of the decays yields the number *N*_blob_ of methacrylate units that can pack inside a *blob*, which is the volume probed by an excited pyrenyl label. Lower *N*_blob_ values are obtained for stiffer chains, that bend less efficiently. The fluorescence decays were acquired in acetonitrile, THF, DMF, and DMSO and the FBM yielded *N*_blob_, which was plotted as a function of pyrene content in Figure 5A–D.

Within experimental error, *N*_blob_ remained constant with pyrene content in Figure 5A–D. *N*_blob_ was averaged over all pyrene contents for all the samples of the same PyEG_5_-PEG_n_MA series in the same solvent to yield <*N*_blob_>, which was plotted as a function of the molecular weight of a structural unit (*MW*(SU)) in Figure 5E–H. The plots shown in Figure 5E–H display some interesting features. For each solvent, <*N*_blob_> was found to decrease with increasing side chain length reflecting the increased extension of the PEG_n_MA backbone with increasing side chain length. <*N*_blob_> reached a plateau value (*N*_blob_^∞^) for the largest side chains indicating that the polymethacrylate backbone appeared fully extended over the length scale probed by an excited pyrenyl label. Finally, <*N*_blob_> for the PyEG_5_-PEG_n_MA samples with an 18-atom-long linker connecting pyrene to the polymethacrylate backbone was significantly larger than <*N*_blob_> obtained earlier for the PyBut-PEG_n_MA samples with a 6-atom-long spacer between pyrene and the polymethacrylate backbone as indicated by the difference between the dashed and solid lines in Figure 5E–H. These differences in <*N*_blob_> between the PyBut-PEG_n_MA and PyEG_5_-PEG_n_MA samples reflect the longer reach of the pyrene derivative used for the latter series.

**Figure 6:**
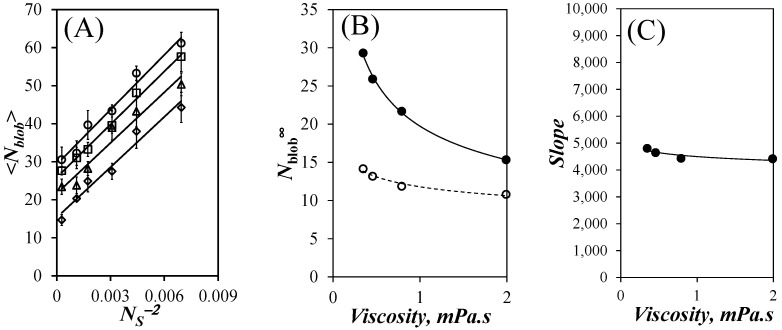
(**A**) Plot of <*N*_blob_> as a function of *N*_S_^−2^ for the PyEG_5_-PEG_n_MA samples except for *n* = 0 in (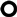) acetonitrile, (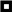) THF, (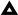) DMF, and (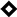) DMSO. (**B**) Plot of *N*_blob_^∞^ as a function of solvent viscosity for the (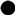) PyEG_5_-PEG_n_MA and (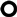) PyBut-PEG_n_MA samples. (**C**) Plot of the slopes of the straight lines in Figure 6A as a function of solvent viscosity.

Another interesting feature in the plots shown in Figure 5E–H was that for the same pyrene content, <*N*_blob_> decreased with increasing solvent viscosity. While this effect had also been observed for the PyBut-PEG_n_MA samples [20], it was much more pronounced for the PyEG_5_-PEG_n_MA samples. This effect could be better visualized in Figure 6A, where <*N*_blob_> was plotted as a function of *N*_S_^−2^, with *N*_S_ being the number of non-hydrogen atoms in the PEG_n_MA side chains equal to 3 + 3 × *n* for a given PEG_n_MA sample. The <*N*_blob_>-*vs*-*N*_S_^−2^ plots in Figure 6A yielded straight lines, except for the <*N*_blob_> value of PyEG_5_-PEG_0_MA, which departed from the linear behavior in all solvents. The largest <*N*_blob_> values were found in acetonitrile, followed by THF, DMF, and DMSO, where the lowest <*N*_blob_> values were obtained. 

The value of <*N*_blob_> for poly(methyl methacrylate) in all solvents was lower than that expected from the straight lines shown in Figure 6A. This is probably because for infinite *N*_S_^−2^, the polymethacrylate backbone still retains some residual stiffness preventing it from collapsing and packing an infinite number of methacrylate monomers when *N*_S_ is infinitely small. Consequently, a limit must be reached experimentally, that prevents <*N*_blob_> from taking an infinite value for infinitely small side chains, as would be otherwise predicted from the straight lines shown in Figure 6A. It is thus reasonable that the <*N*_blob_> values obtained for the PyEG_5_-PEG_n_MA samples having shorter side chains, such as for poly(methyl methacrylate), did not obey the linear <*N*_blob_>-vs-*N*_S_^−2^ found for the PyEG_5_-PEG_n_MA samples with longer side chains in Figure 6A. Extrapolating the straight lines in Figure 6A to the Y-intercept yielded *N*_blob_^∞^ representing the number of methacrylate units encompassed inside a *blob* for a fully extended polymethacrylate backbone. *N*_blob_^∞^ was plotted as a function of solvent viscosity in Figure 6B for the PyEG_5_-PEG_n_MA samples along with the *N*_blob_^∞^ values found earlier for the PyBut-PEG_n_MA samples. The determination of *N*_blob_^∞^ represents the third step in the methodology developed to determine *l*_p_ as shown in Figure 1.

*N*_blob_^∞^ for the PyEG_5_-PEG_n_MA samples was much larger than for the PyBut-PEG_n_MA samples [20] reflecting the longer reach of the linker for the PyEG_5_ derivative [27]. The difference between the *N*_blob_^∞^ values obtained for the PyBut-PEG_n_MA and PyEG_5_-PEG_n_MA samples decreased with increasing viscosity since a larger solvent viscosity hinders the deployment of the pyrenyl labels at the end of the long EG_5_ linker in the PyEG_5_-PEG_n_MA samples during the finite time that the pyrenyl label remains excited. This effect is much less pronounced for the PyBut-PEG_n_MA samples for which the much shorter butyl linker enables the full deployment of the pyrenyl label while it remains excited. Indeed, the *N*_blob_^∞^ dependency on solvent viscosity for the PyBut-PEG_n_MA samples in Figure 6B is much weaker than that for the PyEG_5_-PEG_n_MA samples.

The slopes of the straight lines obtained in Figure 6A were plotted as a function of solvent viscosity in Figure 6C. The slopes showed little dependency on solvent viscosity. The *N*_blob_^∞^ values and the slopes for the PyEG_5_-PEG_n_MA samples could be fitted with power laws, whose empirical expressions are given as Equations (5) and (6), respectively. In turn, the equations for *N*_blob_^∞^ and the slopes could be rearranged to yield the bending function (*f*_b_(*η*,*MW*(*SU*))) in Equation (7). Multiplying *N*_blob_^∞^ by the bending function yielded <*N*_blob_> in Equation (8), which was found to properly describe the experimental <*N*_blob_> values in Figure 5E–H for *MW*(SU) greater than 200 g/mol.

(5) $$N_{blob}^{\infty }\;=\;19.732\times \eta^{-0.365}$$

(6) $$slope\;=\;4498.3\times \eta^{-0.046}$$

(7) $$f_{b}(\eta ,MW(SU))\;=\;1.0\;+\;\frac{227.9698\times \eta^{0.319}}{{\left(3+3\times (MW(SU)-100)/44\right)}^{2}}$$

(8) $$<N_{blob}>\;=\;N_{blob}^{\infty }\times f_{b}(\eta ,MW(SU))$$

The parametrization of the <*N*_blob_> values with Equation (8) could now be applied to predict the persistence length (*l*_p_) for any hypothetical molar mass *MW*(SU) greater than 200 g/mol of a PyEG_5_-PEG_n_MA sample in any solvent as depicted in Figure 2. This was achieved by solving for *l*_p_ in Equation (3) representing the Kratky–Porod equation [24] modified to represent the polymer segment made of <*N*_blob_> structural units of contour length <*N*_blob_> × *b*, where *b* is the length of a methacrylate monomer typically taken to equal 0.25 nm [25,26], inside a *blob* with an end-to-end distance <*r*_EE_^2^>_blob_ [20]. In turn, since all PyEG_5_-PEG_n_MA constructs use the same pyrene derivative, the excited pyrenyl label probes the same volume for all the samples, including those fully extended PyEG_5_-PEG_n_MA samples with infinitely long side chains for which <*r*_EE_^2^>_blob_ is simply equal to *N*_blob_^∞^ × *b*, where the expression of *N*_blob_^∞^ was given in Equation (5).

Since the left-hand side of Equation (3) is known, Equation (3) could be solved to retrieve *l*_p_ for any <*N*_blob_> value obtained with Equation (8) for any solvent viscosity and *MW*(SU) greater than 200 g/mol. The resulting *l*_p_-*vs*-*N*_S_^2^ plots are shown in Figure 6. *l*_p_ increased linearly with increasing *N*_S_^2^ in all solvents considered. The linear increase in *l*_p_ with *N*_S_^2^ agrees with theoretical predictions [33]. The predicted trends obtained with *l*_p_ for the PyEG_5_-PEG_n_MA samples showed a much closer agreement with the experimental data points compared to the trends obtained with *l*_p_ for the PyBut-PEG_n_MA samples, probably because the longer linker of the PyEG_5_ derivative resulted in larger <*N*_blob_> values which were retrieved with better accuracy.

**Figure 7:**
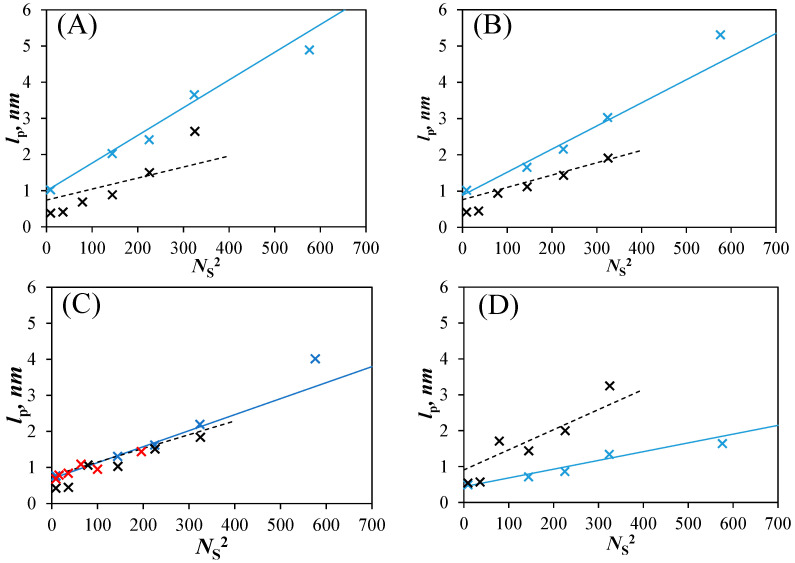
Plot of *l*_p_ as a function of *N*_S_^2^ for the (**×**) PyEG_5_-PEG_n_MA and (**×**) PyBut-PEG_n_MA samples in (**A**) acetonitrile for PyPEG_5_-PEG_n_MA and acetone for PyBut-PEG_n_MA, (**B**) THF, (**C**) DMF, and (**D**) DMSO. (**×**) *l*_p_ values obtained for a series of poly(alkyl methacrylate) [11]. Lines: (solid blue) predicted *l*_p_ values for the PyPEG_5_-PEG_n_MA samples based on Equations (3)–(8) and (dashed black) predicted values for the PyBut-PEG_n_MA samples [20].

As for the *l*_p_ values obtained with the PyBut-PEG_n_MA samples [20], solvent viscosity affected the *l*_p_ values retrieved for the PyEG_5_-PEG_n_MA samples. However, the effect of solvent viscosity on *l*_p_ was opposite between the two polymer series. Whereas an increase in solvent viscosity led to an increase in *l*_p_ for the PyBut-PEG_n_MA samples, it was accompanied by a decrease in *l*_p_ for the PyEG_5_-PEG_n_MA samples. The reason for the opposite trends resided in the different spacers connecting pyrene to the polymethacrylate backbone. In the case of the PyBut-PEG_n_MA samples, the volume of a *blob* (*V*_blob_) was little affected by solvent viscosity [20], as indicated by the small changes in *N*_blob_^∞^ with solvent viscosity observed in Figure 6B. The small dependency of *N*_blob_^∞^ on solvent viscosity enabled the short 6-atom-long spacer to fully deploy, allowing the pyrenyl label to probe a constant *V*_blob_ regardless of solvent viscosity. An increase in solvent viscosity for the PyBut-PEG_n_MA series resulted in weaker PEF, which was erroneously attributed to a stiffening of the chain, resulting in an increase in *l*_p_ based on the Kratky–Porod equation. In contrast, *V*_blob_ was much more strongly affected by solvent viscosity for the longer 18-atom-long penta(ethylene glycol) spacer of the PyEG_5_-PEG_n_MA sample, as illustrated by the significant decrease in *N*_blob_^∞^ in Figure 6B. The inability of the PyEG_5_ derivative to fully deploy while a pyrenyl label remained excited meant that the excited pyrene probed a smaller *V*_blob_ with increasing solvent viscosity. Since the density of a *blob* with a polymer segment of size *N*_blob_ increases with decreasing *N*_blob_ as *N*_blob_/*N*_blob_^3/2^ = *N*_blob_^−0.5^ [15], a smaller *blob* appeared denser, yielding a smaller *l*_p_ for the PyEG_5_-PEG_n_MA samples as observed in Figure 7A–D.

Since effects induced by solvent viscosity on <*N*_blob_> were eliminated when working in a solvent with a viscosity of 0.74 mPa.s approaching that of 0.79 mPa.s for DMF according to molecular mechanics optimizations (MMO) [20], the *l*_p_ values obtained in DMF were expected to best represent the persistence length of PEG_n_MA. As it turned out, the *l*_p_ values retrieved for the PyBut-PEG_n_MA and PyEG_5_-PEG_n_MA samples showed excellent agreement in DMF in Figure 7C. Furthermore, the *l*_p_ values retrieved in DMF matched very closely those reported for a series of poly(alkyl methacrylate)s of similar *MW*(SU) [11], where the poly(alkyl methacrylate)s have been shown to behave similarly to PEG_n_MA over the short length scales probed by an excited pyrenyl label [20]. The concurring trends presented in Figure 7C for the *l*_p_ values retrieved for several polymethacrylates by different procedures provide solid validation of the PEF-based method for measuring the persistence length of these polydisperse polymethacrylate samples.

Another interesting observation in Figure 7C was that the straight lines representing the *l*_p_-*vs*-*N*_S_^2^ trends in Figure 7C did not pass through the origin for an infinitely short side chain. The non-zero Y-intercept is a result of the intrinsic stiffness of the polymethacrylate backbone, which prevents *l*_p_ from reaching zero for an infinitely short side chain. In turn, this conclusion implies that <*N*_blob_> cannot take infinite values for infinitely short side chains as expected from the <*N*_blob_>-*vs*-*N*_S_^−2^ straight lines shown in Figure 6A. Instead, <*N*_blob_> should reach a constant value for shorter side chains approaching the <*N*_blob_> value expected for the unsubstituted polymethacrylate backbone. This most certainly explains why the <*N*_blob_> value for the PyPEG_5_-PEG_0_MA series did not fall on the straight lines in Figure 6A.

**Figure 8:**
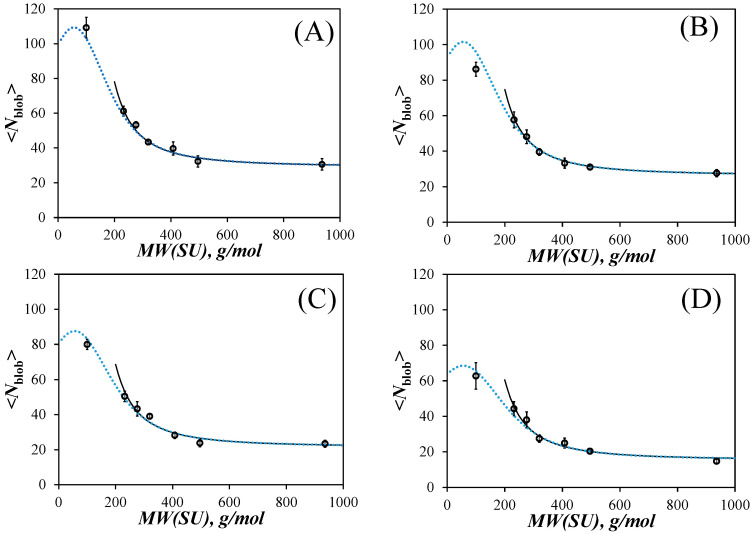
Plots of <*N*_blob_> as a function of *MW*(SU) for the PyEG_5_-PEG_n_MA samples in (**A**) acetonitrile, (**B**) THF, (**C**) DMF, and (**D**) DMSO. Lines: *l*_p_ calculated according to (solid black) Equation (8) and (dotted blue) back calculated from Equation (3).

In order to properly predict the <*N*_blob_> values that should be obtained for polymethacrylates having short side chains, Equations (9) and (10) were used to parametrize the intercepts and slopes of the *l*_p_-*vs*-*N*_S_^2^ straight lines in Figure 6A–D. Combining Equations (9) and (10) yielded the persistence length for any *MW*(SU) and solvent viscosity, which could then be employed with Equation (3) to extract <*N*_blob_>. The resulting plots of <*N*_blob_> as a function of *MW*(SU) are shown in Figure 8A–D for acetonitrile, THF, DMF, and DMSO.

The new trends pass through most of the data points including the <*N*_blob_> value for PyEG_5_-PEG_0_MA. Instead of <*N*_blob_> tending to infinity for infinitely small side chains, <*N*_blob_> goes through an inflection point as it approaches *MW*(SU) = 100 g/mol for poly(methyl methacrylate) before passing through a maximum and intercepting the *Y*-axis at a finite, non-zero value. The small dip observed for short side chains before the maximum in the plots of Figure 8 is certainly an artefact resulting from the mathematical handling of the Kratky–Porod equation with the linear relationship between *l*_p_ and *N*_S_^2^ in Figure 7 to predict <*N*_blob_>. Despite this mathematical artefact, the plots of <*N*_blob_>-*vs*-*MW*(SU) in Figure 8 provide a physically more realistic depiction of the behavior of <*N*_blob_> expected as the side chains become infinitely short, because <*N*_blob_> no longer diverges to infinity as would be otherwise predicted with Equation (8).

*l*_p_(intercept) = 0.610 × *η*^−0.469^ (9)

*l*_p_(slope) = 3.82 × 10^−3^ × *η*^−0.654^ (10)

**Figure 9:**
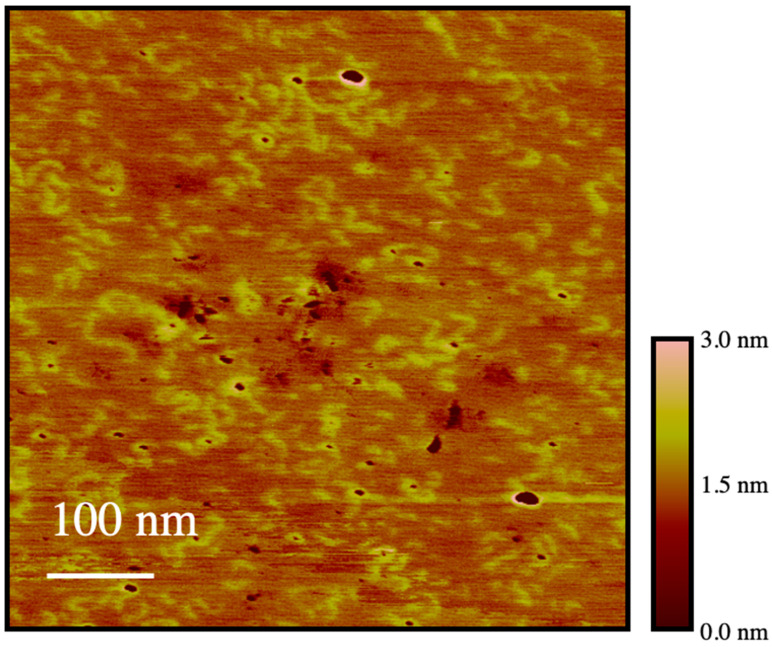
AFM topography image of PEG_19_MA spin-coated on a freshly cleaved mica surface from a 10 mg/L solution in THF.

The backbone conformation of PEG_19_MA was further investigated by atomic force microscopy (AFM) to visualize individual PEG_19_MA macromolecules, which were prepared without pyrene. This sample had a number (*M*_n_) and weight (*M*_w_) average molecular weight of 134,000 and 193,000 g·mol^−1^, respectively. Individual polymer molecules were observed in Figure 9 ranging in length from 20 to 90 nm, in diameter from 10 to 20 nm, and in height from 0.5 to 1 nm. These results are consistent with the expected dimensions of these macromolecules considering that a fully extended PEG_19_MA macromolecule would have a number average contour length of ~35 nm and an average width of ~16 nm.

Furthermore, the AFM image shown in Figure 9 clearly demonstrates the presence of isolated macromolecules with no indication of aggregation. This observation eliminates the possibility that PEG_19_MA could aggregate, as has been found for PBBs prepared with longer poly(ethylene oxide) side chains, which have been shown to crystalize resulting in the formation of crystalsomes [34]. Figure 9 demonstrates that this is not the case for PEG_19_MA. Although the chains observed in Figure 9 show some curvature, the steric hindrance generated by their side chains prevents them from adopting a fully coiled conformation.

The image shown in Figure 9 complements the conclusions drawn from the plateau reached for *m*(*I*_E_/*I*_M_) in Figure 4 and for *N*_blob_ in Figure 5E–H obtained by steady-state and time-resolved fluorescence, respectively. These plateaus were defined mostly by the *m*(*I*_E_/*I*_M_) and *N*_blob_ values obtained with samples from the PyEG_5_-PEG_19_MA series, and they were rationalized by evoking the stiffening and extension of the polymer backbone. Such a stiffening is clearly visible in the AFM picture where the PEG_19_MA macromolecules appear as WLCs.

## 4. Conclusions

The PEF-based method introduced earlier to determine the persistence length of polymers [20] that are polydisperse was improved by widening the volume probed by an excited pyrenyl label. This was achieved by using a penta(ethylene glycol) 1-pyrenemethyl ether derivative to generate an 18-atom-long spacer between the pyrenyl label and the polymethacrylate backbone instead of the sort 6-atom-long spacer obtained with the commercially available 1-pyrenebutanol derivative [20]. Considering that *N*_blob_^∞^ ranged from 15 in DMSO to 29 in acetonitrile for the PyEG_5_-PEG_n_MA samples, an excited pyrenyl label could probe a *blob*, whose diameter equal to *N*_blob_^∞^ × *b* ranged between 3.8 nm in DMSO and 7.3 nm in acetonitrile, significantly larger than the *blobs* with a 3.0 nm diameter obtained with the PyBut-PEG_n_MA samples [20]. The larger *blobs* probed with the PyEG_5_-PEG_n_MA samples enabled larger persistence lengths to be measured, reaching up to 4.0 nm compared to the maximum persistence length of 2.0 nm reached earlier with the PyBut-PEG_n_MA samples. Excellent agreement was obtained for the persistence lengths determined with the PyBut-PEG_n_MA and PyEG_5_-PEG_n_MA samples in DMF, a solvent whose viscosity happens to eliminate the effects induced on <*N*_blob_> by solvent viscosity. By spreading <*N*_blob_> over a much wider range of *MW*(SU) values in Figure 5E–H, three regimes could be clearly identified for different lengths of side chains in Figure 8. On the one hand, short side chains yield <*N*_blob_> values that change little with side chain length as <*N*_blob_> reflects the intrinsic flexibility of the unsubstituted polymethacrylate backbone. On the other hand, very long side chains induce a fully extended conformation of the PEG_n_MA backbone resulting in <*N*_blob_> taking a constant value with side chain length equal to *N*_blob_^∞^. Significant changes in <*N*_blob_> are only observed for PEG_n_MA samples with intermediate side chain lengths between two and nine ethylene glycol units. In turn, the range of <*N*_blob_> values is directly related to the range of persistence lengths that can be retrieved from PEF measurements conducted with PEG_n_MA samples labeled with a given pyrene derivative, as shown with the plots in Figure 8. Consequently, this study confirms the potential of PEF-based methods to characterize the persistence length of polydisperse polymers in any organic solvent, which should enable the determination of the unknown persistence length of many polymers.

## Figures and Tables

**Table 1:** Chemical structure of the PyEG_5_-PEG_n_MA samples with the number of atoms (*N*_S_) in each side chain and the molecular weight of the structural unit (*MW*(SU)).

Sample	PyEG_5_-PEG_0_MA	PyEG_5_-PEG_3_MA	PyEG_5_-PEG_4_MA	PyEG_5_-PEG_5_MA	PyEG_5_-PEG_7_MA	PyEG_5_-PEG_9_MA	PyEG_5_-PEG_19_MA
Structure	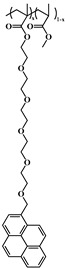	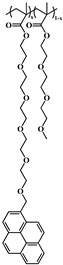	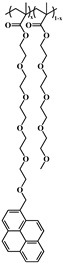	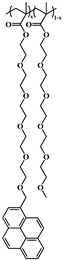	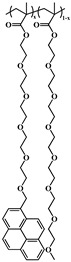	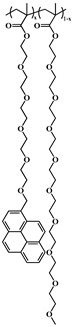	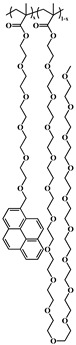
*N*_S_	3	12	15	18	24	30	60
*MW*(SU) g/mol	100	232	276	320	408	500	950

**Table 2:** Pyrene content, absolute *M*_n_, and dispersity of the PyEG_5_-PEG_n_MA samples.

PyEG_5_-PEG_0_MA			PyEG_5_-PEG_3_MA			PyEG_5_-PEG_4_MA		
Pyrene Content (mol%)	*M*_n_ (g/mol) ^a^	*Ð*	Pyrene Content (mol%)	*M*_n_ (g/mol) ^a^	*Ð*	Pyrene Content (mol%)	*M*_n_ (g/mol) ^a^	*Ð*
0.9	40,000	1.6	0	293,000	2.9	1.2	105,000	1.5
2.0	45,000	1.3	0.5	153,000	2.7	1.9	84,000	1.6
2.6	24,000	1.3	1.6	118,000	1.9	2.2	88,000	1.5
3.3	43,000	1.8	2.0	128,000	2.8	2.4	83,000	1.4
3.3	46,000	1.5	2.0	149,000	3.0	3.1	89,000	1.3
-	-	-	3.0	200,000	1.7	-	-	-
-	-	-	3.4	91,000	2.1	-	-	-
-	-	-	3.8	150,000	1.6	-	-	-
**PyEG_5_-PEG_5_MA**			**PyEG_5_-PEG_7_MA**			**PyEG_5_-PEG_9_MA**		
**Pyrene Content (mol%)**	***M*_n_ (g/mol) ^b^**	***Ð***	**Pyrene Content (mol%)**	***M*_n_ (g/mol) ^b^**	***Ð***	**Pyrene Content (mol%)**	***M*_n_ (g/mol) ^b^**	***Ð***
0.4	121,000	2.1	2.2	232,200	3.8	0	152,000	1.5
2.2	55,000	1.6	3.0	222,000	3.7	0.8	420,000	3.4
3.6	95,000	2.0	3.6	304,000	3.5	2.9	257,000	1.5
3.8	86,000	1.7	4.2	109,000	1.8	5.2	166,000	1.6
4.8	106,000	2.3	-	-	-	5.8	154,000	1.3
6.5	64,000	1.5	-	-	-	7.4	173,000	1.4
-	-	-	-	-	-	10.2	186,000	1.6
**PyEG_5_-PEG_19_MA**								
**Pyrene Content (mol%)**	***M*_n_ (g/mol) ^b^**	***Ð***						
0	134,000	1.4						
0.9	187,000	1.2						
2.1	194,000	1.3						
3.2	683,000	2.8						
4.1	132,000	2.1						
5.2	85,300	1.3						
6.2	274,000	1.5						
6.6	92,000	1.3						

^a^ GPC in THF. ^b^ GPC in DMSO.

## Data Availability

Data will be made available upon request.

## Supplementary Materials

The following supporting information can be downloaded at: https://www.mdpi.com/article/10.3390/polym15193958/s1, Equations used in the FBM analysis, Figure S1. ^1^H NMR spectrum of penta(ethylene glycol) mono *p*-toluene sulfonate, Figure S2. ^1^H NMR spectrum of 1-pyrenemethyl ether penta(ethylene glycol), Figure S3. ^1^H NMR spectrum of penta(ethylene glycol) methyl ether methacrylate, Figure S4. ^1^H NMR spectrum of PyEG_5_(3.6)-PEG_5_MA, Figure S5. GPC traces in THF with DRI and absorption detector for the representative PyEG_5_-PEG_n_MA samples, Figure S6. SSF spectra of PyEG_5_-PEG_n_MA samples in acetonitrile, Figure S7. SSF spectra of PyEG_5_-PEG_n_MA samples in THF, Figure S8. SSF spectra of PyEG_5_-PEG_n_MA samples in DMF, Figure S9. SSF spectra of PyEG_5_-PEG_n_MA samples in DMSO, Figure S10. Plot of *I*_E_/*I*_M_ ratio versus pyrene content for PyEG_5_-PEG_n_MA in acetonitrile, THF, DMF, and DSMO, Figure S11. Global FBM analysis of the monomer and excimer fluorescence decays for the PyEG_5_(2.6)-PEG_0_MA, Tables S1–S12. Parameters retrieved from the FBM analysis of the monomer and excimer decays for the PyEG_5_-PEG_n_MA samples in acetonitrile, THF, DMF, and DMSO.
