# Supplementary material for: Persistence Length of PEGMA Bottle Brushes Determined by Pyrene Excimer Fluorescence
Source: Polymers (Basel). 2023 Sep 30;15(19):3958. doi: 10.3390/polym15193958 (PMC10575010; doi:10.3390/polym15193958)
Supplement: Supplementary file 1 [file polymers-15-03958-s001.zip › polymers-2628977-supplementary.pdf]

Supporting Information for

Persistence Length of PEGMA Bottle Brushes Determined by Pyrene Excimer Fluorescence

Janine L. Thoma, Hunter Little, Jean Duhamel*

Institute for Polymer Research, Waterloo Institute for Nanotechnology, Department of Chemistry, University of Waterloo, Waterloo, ON N2L 3G1, Canada

Lei Zhang, Kam Tong Leung

WATLab and Department of Chemistry, University of Waterloo, Waterloo, ON N2L3G1, Canada

* To Whom correspondence should be addressed.

Jean Duhamel: jduhamel@uwaterloo.ca

Kam Tong Leung : tong@uwaterloo.ca

Table of Contents

A] *Fluorescence Blob Model (FBM) Equations*……………………………………………….. 3-4

B] *^{1}H NMR spectra*…………………………………………………………..………… 5-8

C] *Gel permeation chromatography* ……….………………………………………………… 9-10

D] *Steady-state fluorescence spectra and I_E/I_M plots*……………………………………... 11-15

E] *Sample fit of the global FBM analysis of the fluorescence decays* …………………………..16

F] *Parameters retrieved from the FBM analysis*…………………………………………… 17-28

G] *References*…………………………………………………………………………..……. 29

A] Equations used to analyze the fluorescence decays of the PyEG5-PEGnMA samples

Equations S1 and S2 were employed to globally fit the monomer and excimer fluorescence decays of the PyEG5-PEGnMA samples, respectively.[1,2]

$$[Py^*]_{(t)} = [Py_{diff}^*]_{(t)} + [Py_{k2}^*]_{(t)} + [Py_{free}^*]_{(t)} = [Py_{diff}]_o \exp\left(-\left(A_2 + \frac{1}{\tau_M}\right)t - A_3\left(1-\exp(-A_4 t)\right)\right)$$

$$+\left([Py_{k2}]_o + [Py_{diff}]_o e^{-A_3} \sum_{i=0}^{\infty} \frac{A_3^i}{i!} \frac{A_2 + iA_4}{A_2 + iA_4 - k_2}\right) \exp\left(-\left(k_2 + \frac{1}{\tau_M}\right)t\right)$$

$$-[Py_{diff}]_o e^{-A_3} \sum_{i=0}^{\infty} \frac{A_3^i}{i!} \frac{A_2 + iA_4}{A_2 + iA_4 - k_2} \exp\left(-\left(A_2 + iA_4 + \frac{1}{\tau_M}\right)t\right)$$

$$+[Py_{free}]_o \exp\left(-\frac{t}{\tau_M}\right)\Bigg] \qquad \text{(S1)}$$

$$[E^*]_{(t)} = [E0^*]_{(t)} + [D^*]_{(t)} = k_2\left(\left([Py_{k2}(E0)]_o + [Py_{diff}(E0)]_o e^{-A_3} \sum_{i=0}^{\infty} \frac{A_3^i}{i!} \frac{A_2 + iA_4}{A_2 + iA_4 - k_2}\right)\right.$$

$$\times \frac{\exp\left(-\frac{t}{\tau_{E0}}\right) - \exp\left(-\left(k_2 + \frac{1}{\tau_M}\right)t\right)}{k_2 + \frac{1}{\tau_M} - \frac{1}{\tau_{E0}}}$$

$$\left. + [Py_{diff}(E0)]_o e^{-A_3} \sum_{i=0}^{\infty} \frac{A_3^i}{i!} \frac{A_2 + iA_4}{A_2 + iA_4 - k_2} \frac{\exp\left(-\left(A_2 + iA_4 + \frac{1}{\tau_M}\right)t\right) - \exp\left(-\frac{t}{\tau_{E0}}\right)}{A_2 + iA_4 + \frac{1}{\tau_M} - \frac{1}{\tau_{E0}}}\right)$$

$$+k_2\left(\left([Py_{k2}(D)]_o+[Py_{diff}(D)]_o e^{-A_3}\sum_{i=0}^{\infty}\frac{A_3^i}{i!}\frac{A_2+iA_4}{A_2+iA_4-k_2}\right)\times\frac{\exp\left(-\frac{t}{\tau_D}\right)-\exp\left(-\left(k_2+\frac{1}{\tau_M}\right)t\right)}{k_2+\frac{1}{\tau_M}-\frac{1}{\tau_D}}\right.$$

$$\left.+[Py_{diff}(D)]_o e^{-A_3}\sum_{i=0}^{\infty}\frac{A_3^i}{i!}\frac{A_2+iA_4}{A_2+iA_4-k_2}\frac{\exp\left(-\left(A_2+iA_4+\frac{1}{\tau_M}\right)t\right)-\exp\left(-\frac{t}{\tau_D}\right)}{A_2+iA_4+\frac{1}{\tau_M}-\frac{1}{\tau_D}}\right)$$

$$\left.+[E0]_o\times\exp\left(-\frac{t}{\tau_{E0}}\right)+[D]_o\times\exp\left(-\frac{t}{\tau_D}\right)\right] \qquad \text{(S2)}$$

In Equations S1 and S2, the parameters A_2, A_3, and A_4 are given in Equations S3.a–c.

$$A_2 = <n>\times\frac{k_{blob}k_e[blob]}{k_{blob}+k_e[blob]} \qquad \text{(S3.a)}$$

$$A_3 = <n>\times\left(\frac{k_{blob}}{k_{blob}+k_e[blob]}\right)^2 \qquad \text{(S3.b)}$$

$$A_4 = k_{blob}+k_e[blob] \qquad \text{(S3.c)}$$

B] ^{1}H NMR spectra

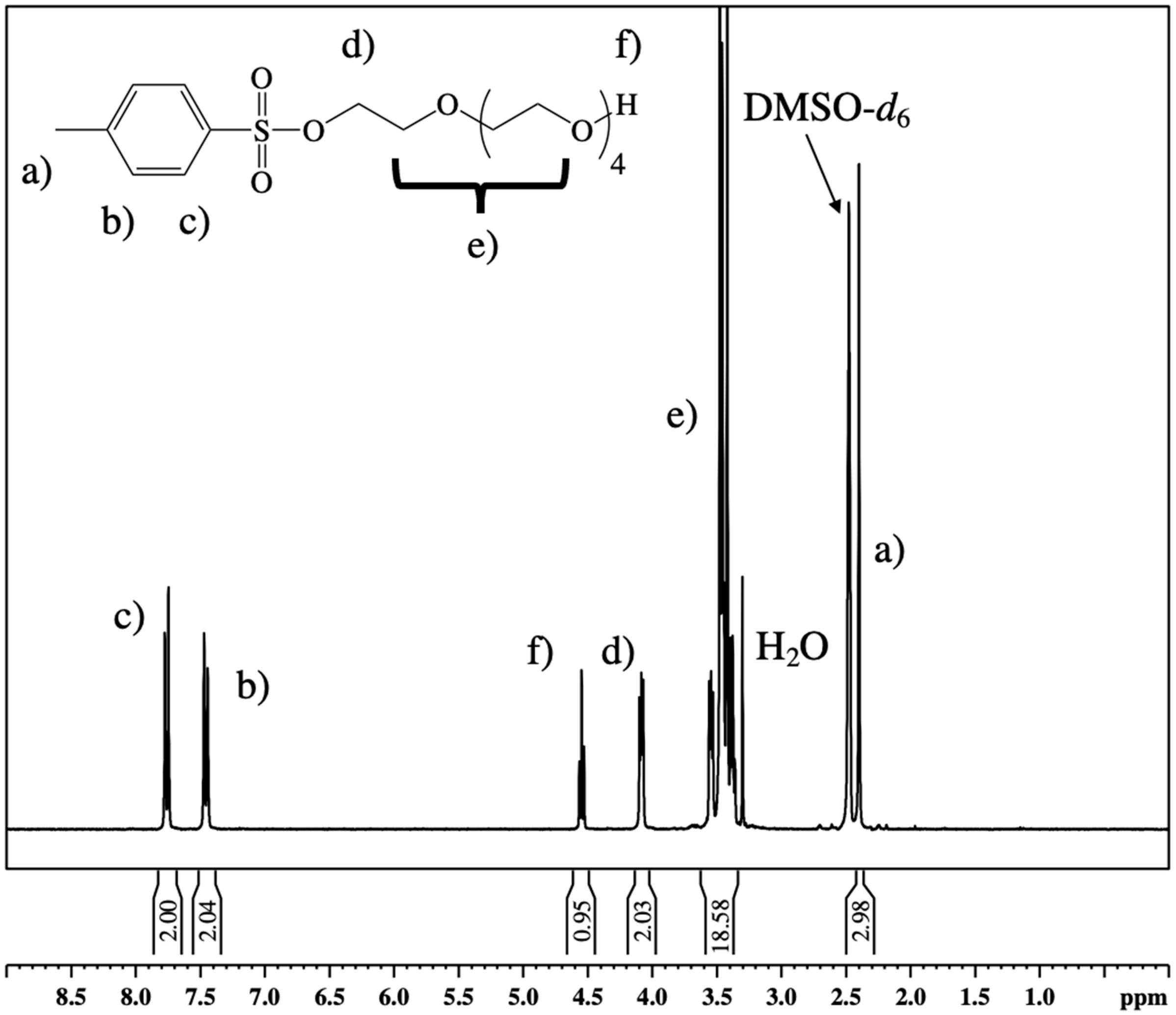

Figure S1. ^{1}H NMR spectrum of penta(ethylene glycol) mono *p*-toluene sulfonate. (DMSO-d_6, 300 MHz), δ 2.40 (s, 3H), 3.34-3.59 (m, 18H), 4.08 (t, 2H), 4.54 (t, 1H), 7,44 (d, 2H), 7.76 (d, 2H).

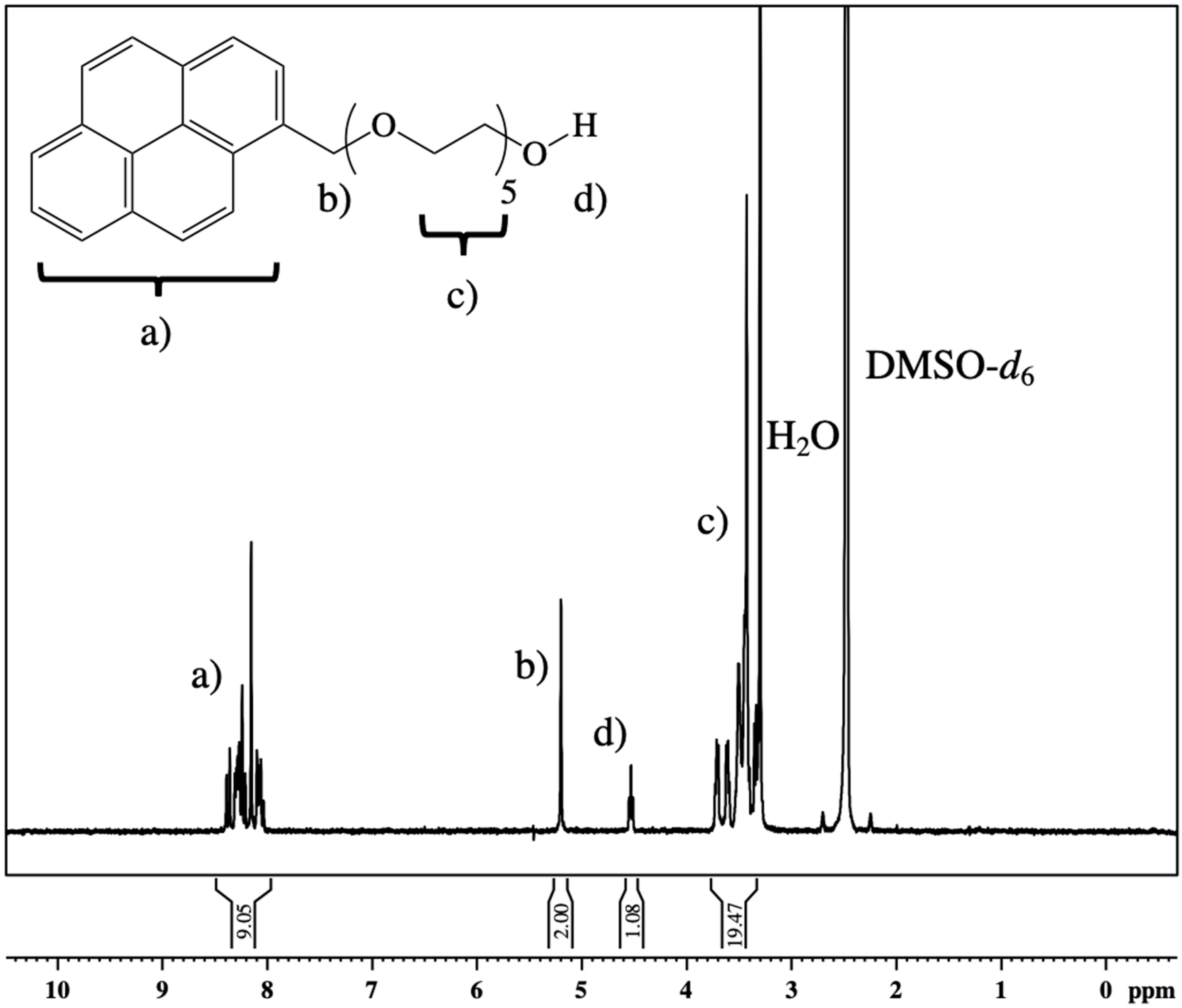

Figure S2. ^{1}H NMR spectrum of 1-pyrenemethyl ether penta(ethylene glycol) (DMSO-d_6, 300 MHz), δ 3.33-3.74 (m, 20H), 4.53 (t, 1H), 5.20 (s, 2H), 8.02-8.41 (m, 9H).

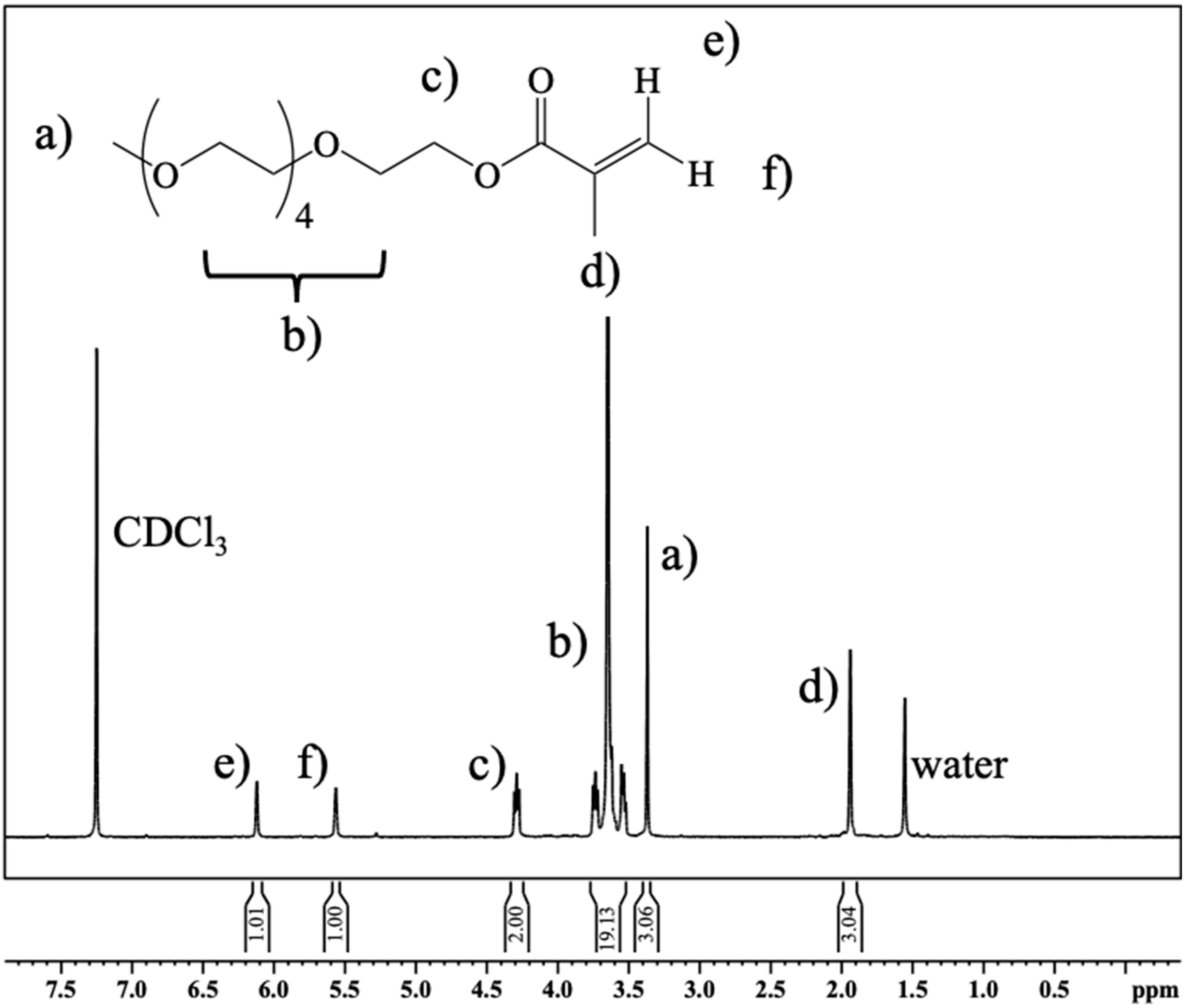

Figure S3. ^{1}H NMR spectrum of penta(ethylene glycol) methyl ether methacrylate ($CDCl_3$, 300 MHz), δ 1.94 (s, 3H), 3.37 (s, 3H), 3.51-3.77 (m, 18H), 4.29 (t, 2H), 5.57 (s, 1H), 6.13 (s,1H).

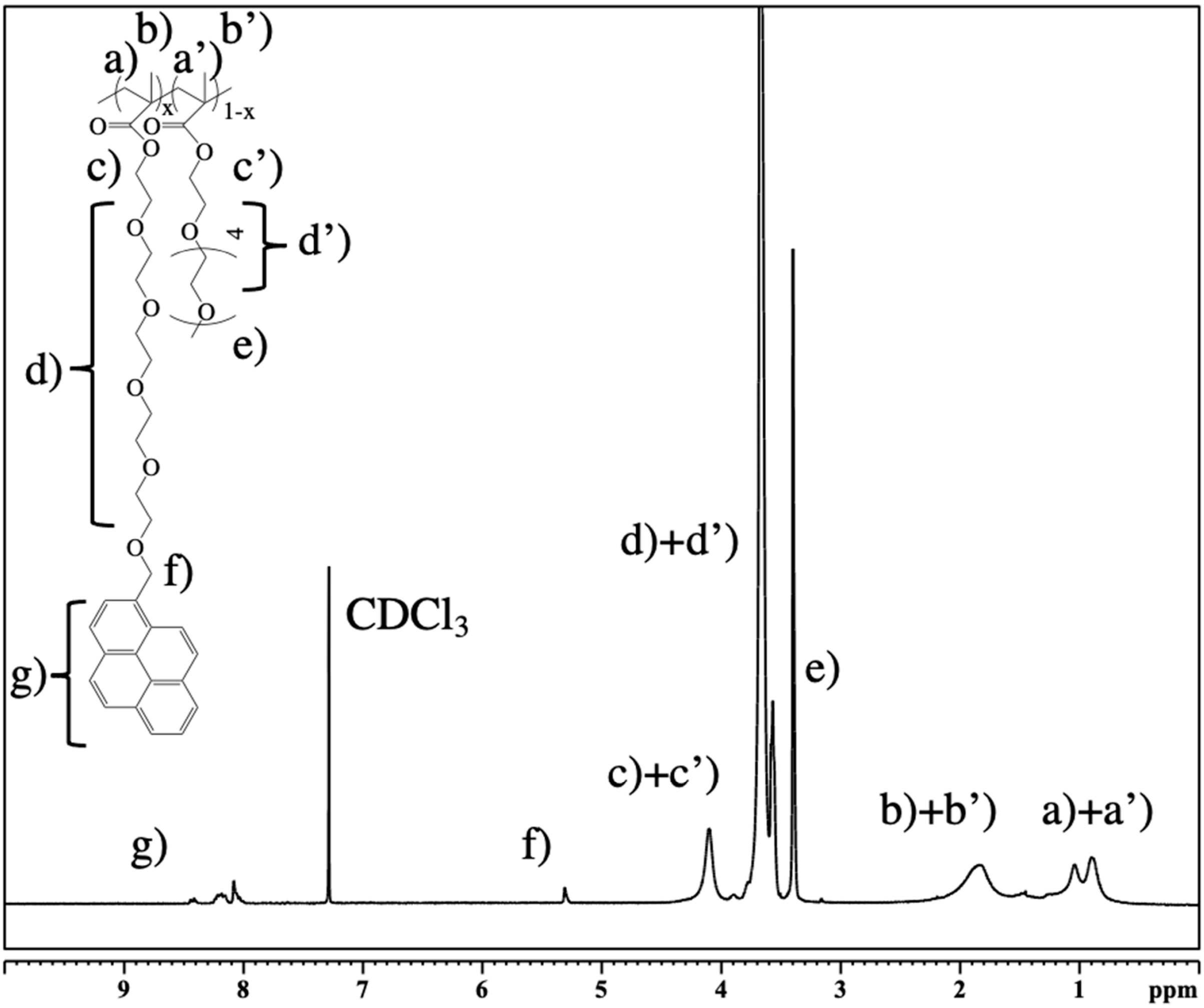

Figure S4. ^{1}H NMR spectrum of PyEG$_5$(3.6)-PEG$_5$MA. ($CDCl_3$, 300 MHz).

C] Gel permeation chromatography

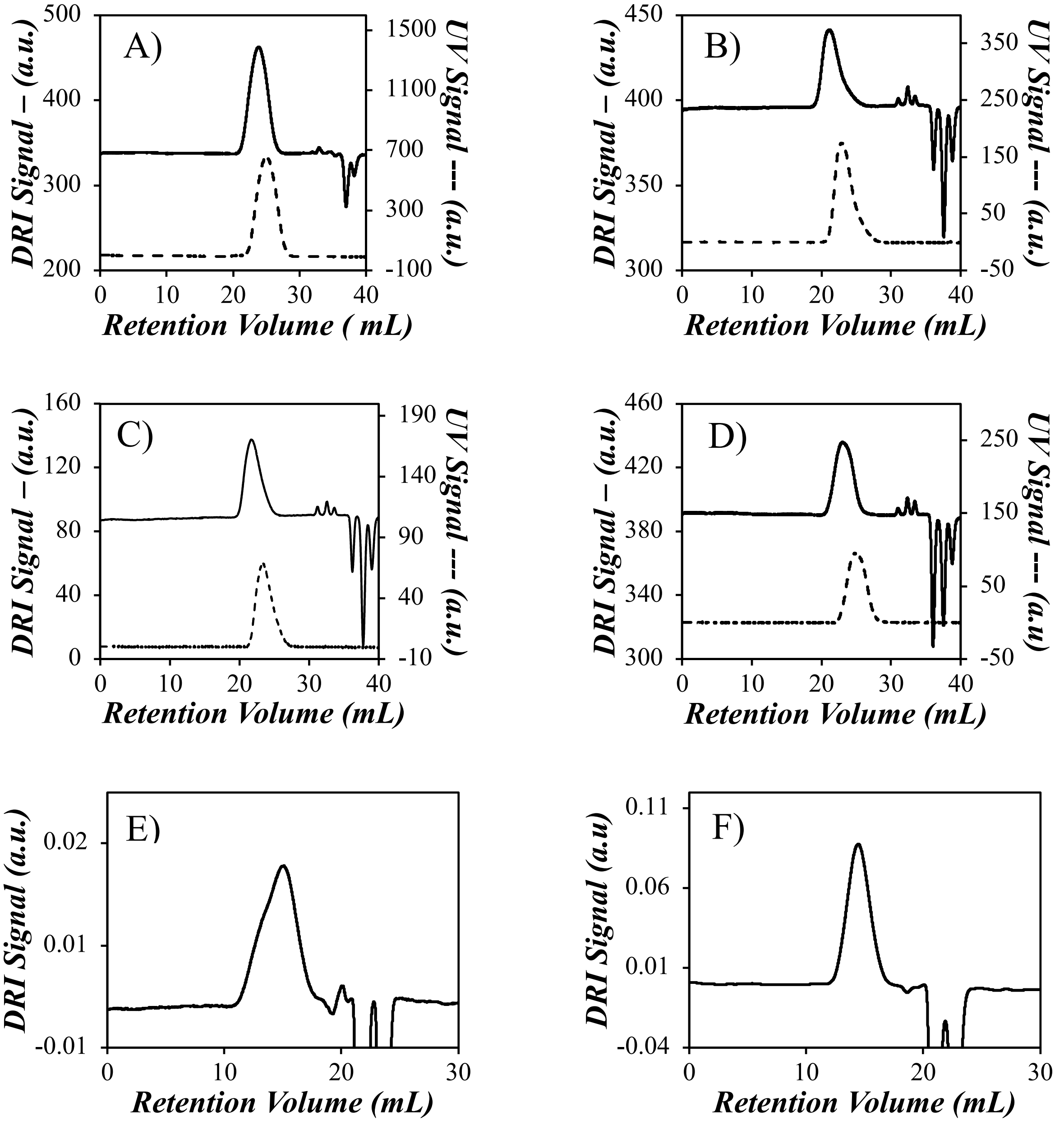

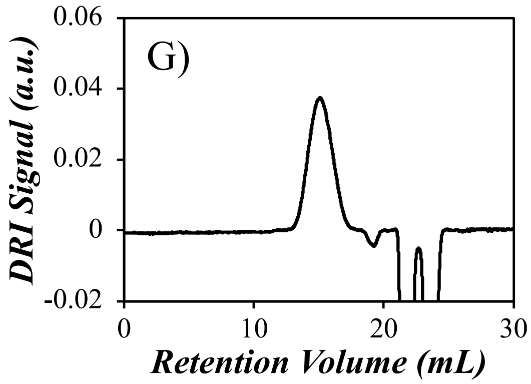

Figure S5. GPC traces in THF with (———) DRI and (- - - - -) absorption detector for A) $PyEG_5$(2.6)-PEG_0MA, B) $PyEG_5$(3.4)-PEG_3MA, C) $PyEG_4$(1.2)-PEG_5MA and D) $PyEG_5$(2.2)-PEG_5MA. GPC traces in DMSO with DRI detector only for E) $PyEG_5$(3.6%)-PEG_7MA, F) $PyEG_5$(5.2)-PEG_9MA, and G) $PyEG_5$(2.1)-$PEG_{19}MA$. The number in parenthesis indicates the molar percentage of pyrene-labeled structural units.

All samples run in DMSO were first injected into a GPC instrument, that used *N*,*N*-dimethylformamide (DMF) with 0.1 wt% LiCl as eluent. The instrument was equipped with a DRI and UV detector. This was done to ensure that the polymers were free of any unreacted monomer or free pyrene derivative. Since the GPC system in DMF was not equipped with a light scattering detector, the polymers were re-injected into the DMSO GPC for absolute molecular weight determination.

D] Steady-state fluorescence spectra

The SSF spectra for each PyEG$_5$-PEG$_n$MA PBB in degassed tetrahydrofuran (THF), acetonitrile, dimethylformamide (DMF), and dimethylsulfoxide (DMSO).

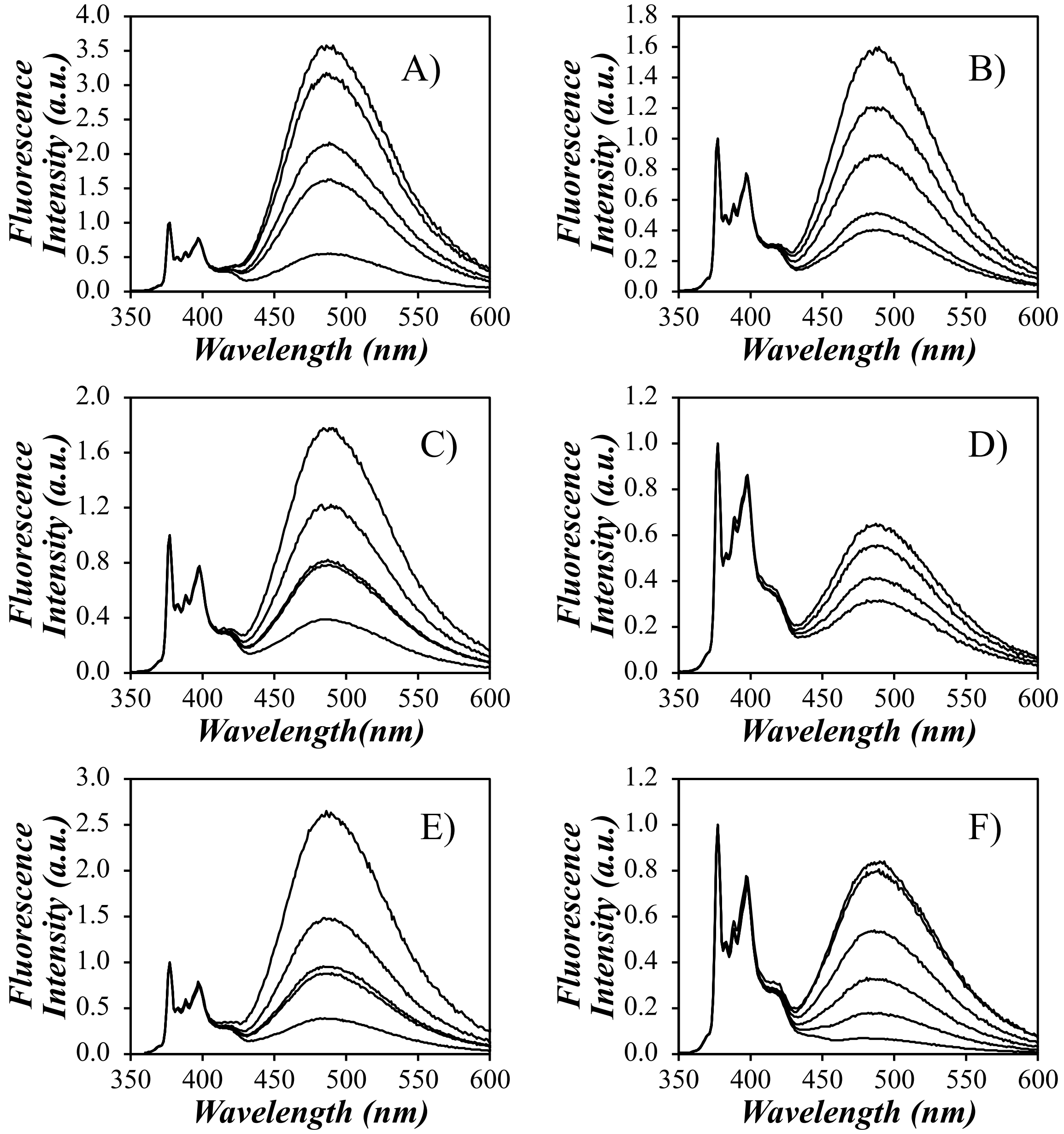

Figure S6. SSF spectra of A) PyEG$_5$-PEG$_0$MA, B) PyEG$_5$-PEG$_3$MA, C) PyEG$_5$-PEG$_5$MA, D) PyEG$_5$-PEG$_7$MA, E) PyEG$_5$-PEG$_9$MA, and F) PyEG$_5$-PEG$_{19}$MA in acetonitrile. λ_{ex}=344 nm.

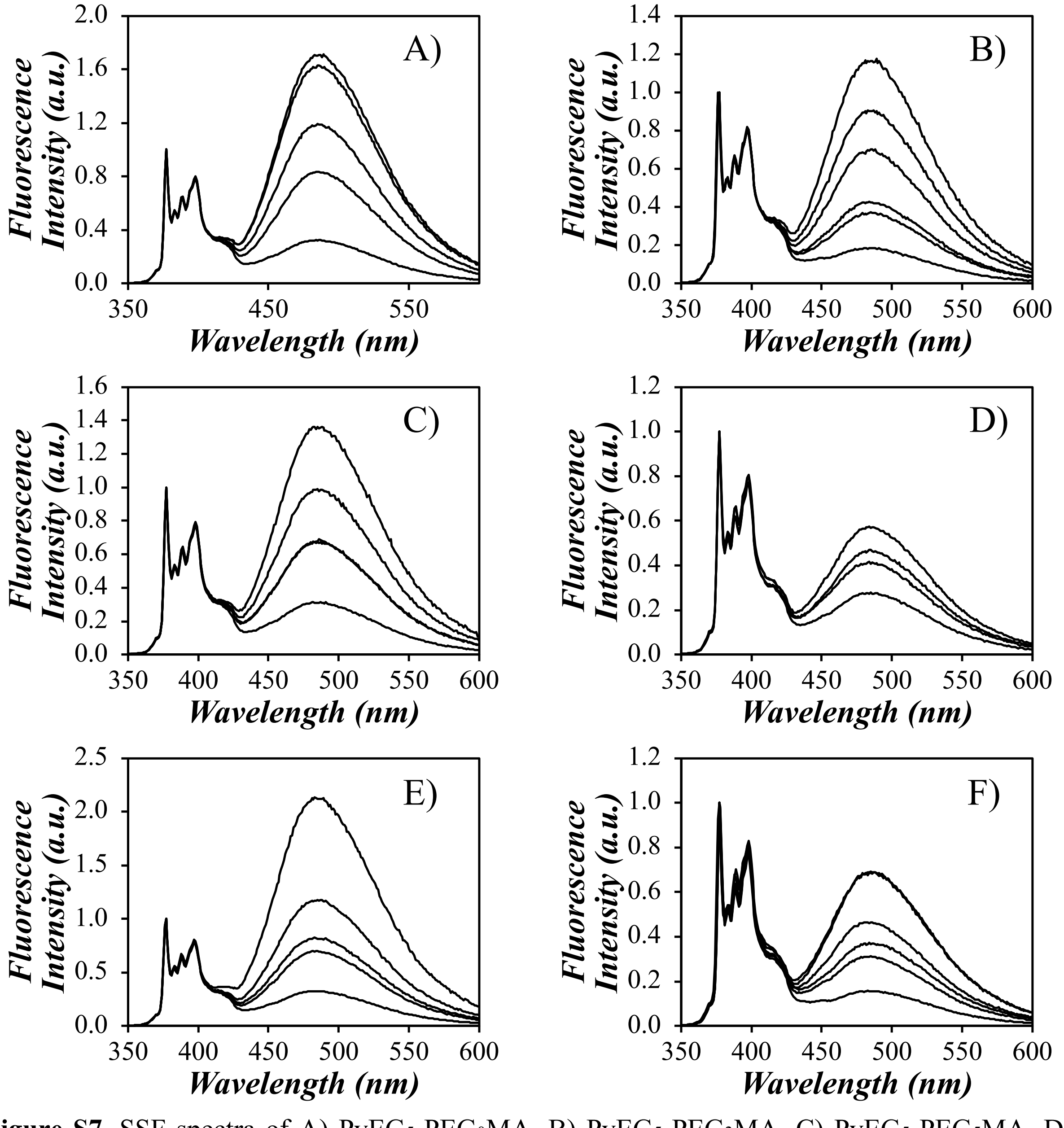

Figure S7. SSF spectra of A) PyEG$_5$-PEG$_0$MA, B) PyEG$_5$-PEG$_3$MA, C) PyEG$_5$-PEG$_5$MA, D) PyEG$_5$-PEG$_7$MA, E) PyEG$_5$-PEG$_9$MA, and F) PyEG$_5$-PEG$_{19}$MA in THF. λ_{ex}=344 nm.

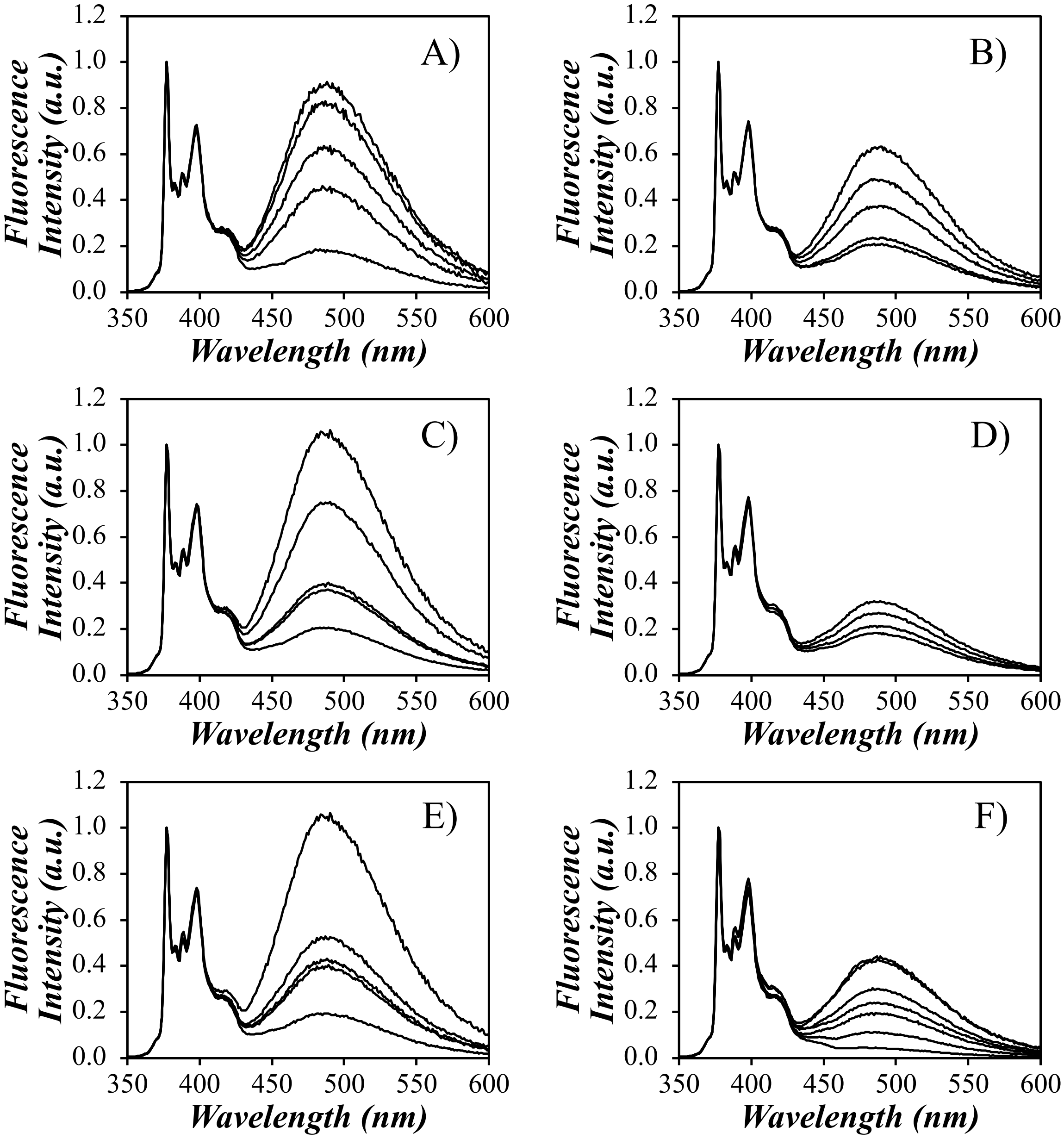

Figure S8. SSF spectra of A) $PyEG_5$-PEG_0MA, B) $PyEG_5$-PEG_3MA, C) $PyEG_5$-PEG_5MA, D) $PyEG_5$-PEG_7MA, E) $PyEG_5$-PEG_9MA, and F) $PyEG_5$-$PEG_{19}MA$ in DMF. λ_{ex}=344 nm.

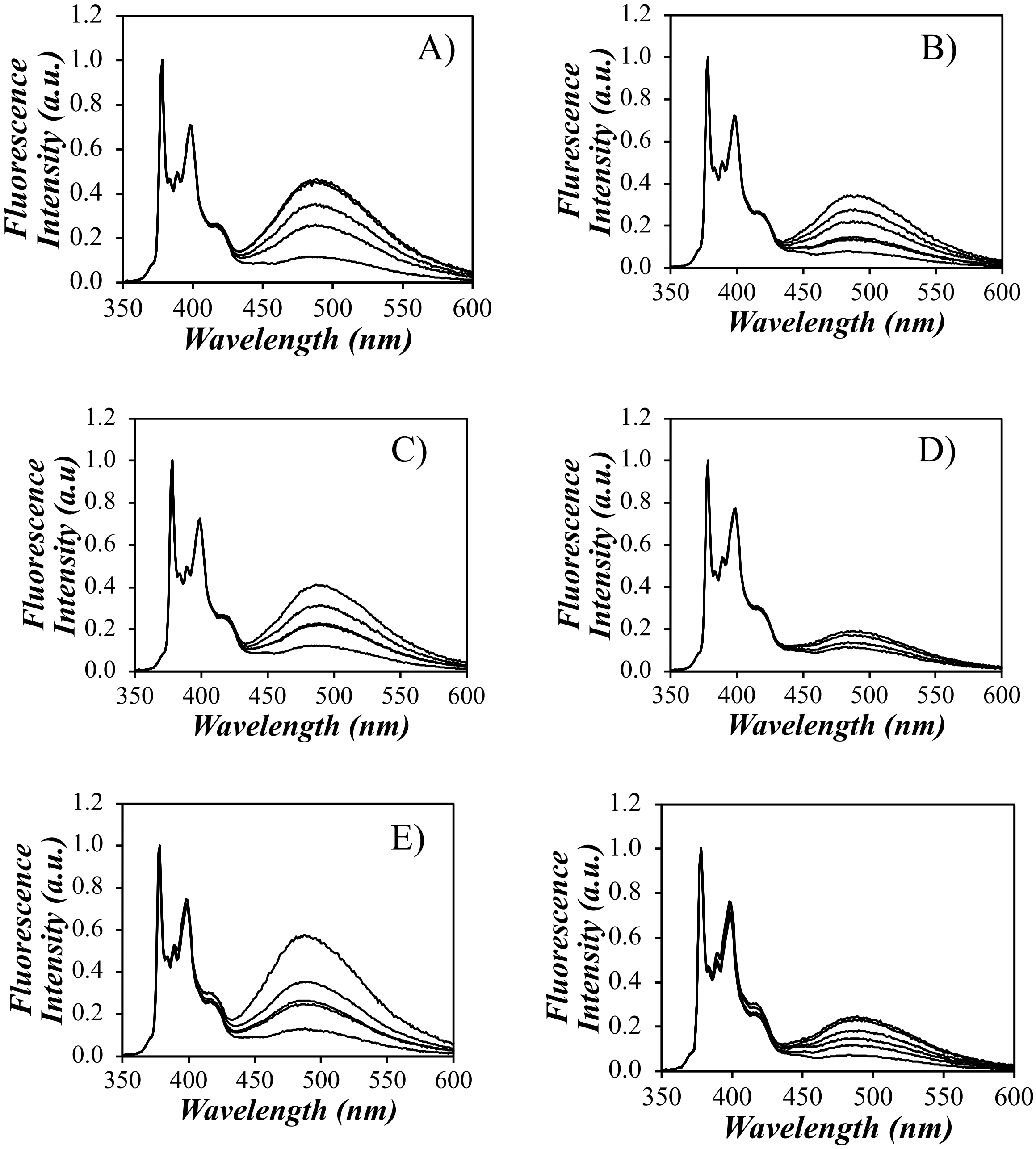

Figure S9. Steady-state fluorescence spectra of A) PyEG$_5$-PEG$_0$MA, B) PyEG$_5$-PEG$_3$MA, C) PyEG$_5$-PEG$_5$MA, D) PyEG$_5$-PEG$_7$MA, E) PyEG$_5$-PEG$_9$MA, and F) PyEG$_5$-PEG$_{19}$MA in DMSO. [*Py*] = 2.5×10^{-6} M; λ_{ex} = 344 nm.

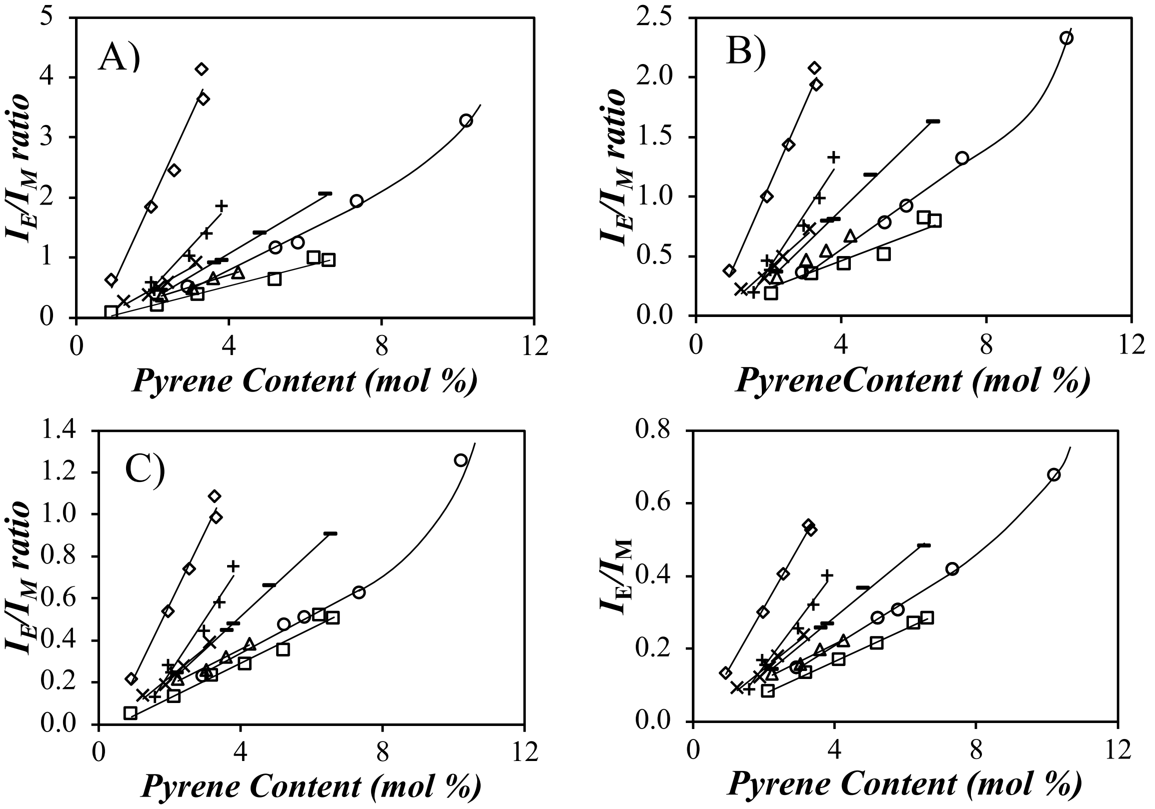

Figure S10. Plot of I_E/I_M ratio versus pyrene content for $PyEG_5$-PEG_nMA in A) acetonitrile, B) THF, C) DMF, and D) DMSO. (◆) $PyEG_5$-PEG_0MA, (+) $PyEG_5$-PEG_3MA, (×) $PyEG_5$-PEG_4MA, (–) $PyEG_5$-PEG_5MA, (▲) $PyEG_5$-PEG_7MA, (●) $PyEG_5$-PEG_9MA, and (■) $PyEG_5$-$PEG_{19}MA$.

E] Sample fit of the global FBM analysis of the fluorescence decays

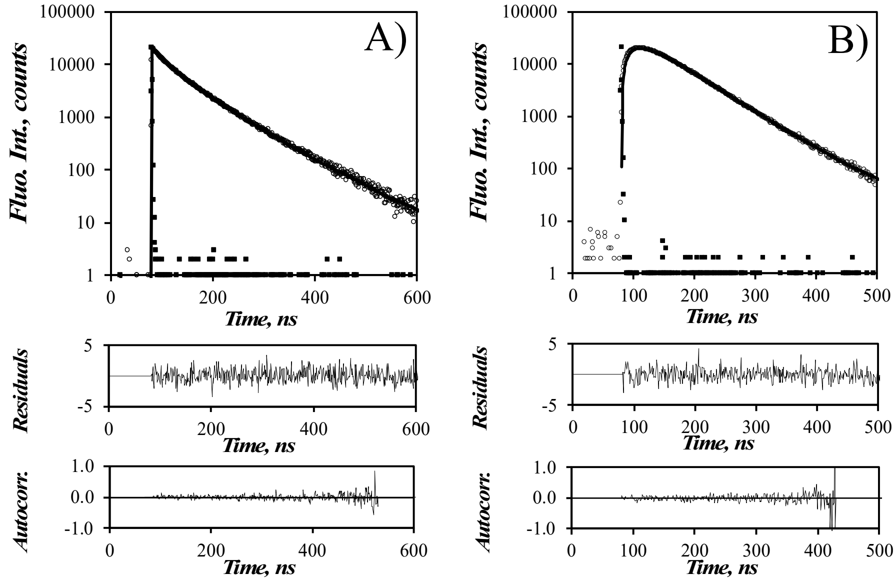

Figure S11. Global FBM analysis of the (A) monomer (λ_{em} = 375 nm) and (B) excimer (λ_{em} = 510 nm) fluorescence decays for PyEG$_5$-PEG$_0$MA with a pyrene content of 2.6 mol %. χ^2 = 1.15, λ_{ex} = 344 nm.

F] Parameters retrieved from the FBM analysis

Table S1. Parameters retrieved from the FBM analysis of the monomer decays for the PyEG$_5$-PEG$_n$MA samples in degassed acetonitrile.

Sample	Mol%	k_{blob} (10^6s^{-1})	<n>	$k_e[blob]$ (10^6s^{-1})	f_{Mdiff}	f_{k2}	f_{Mfree}	f_{MS}	χ^2
	0.9	11.4	1.39	4.34	0.68	0.24	0.08	-	1.0
PyEG$_5$-PEG$_0$MA	2.0	11.8	2.30	3.50	0.57	0.42	0.02	-	1.2
$k_2 = 1.49\times10^8$ s^{-1}	2.6	12.0	2.70	3.65	0.46	0.53	0.01	-	1.1
τ_M = 270 ns	3.3	10.7	3.71	2.42	0.37	0.62	0.00	-	1.2
	3.3	10.8	3.47	2.37	0.38	0.61	0.00	-	1.1
	2.0	12.2	1.28	4.33	0.63	0.29	0.08	-	1.0
PyEG$_5$-PEG$_3$MA	2.1	12.3	1.09	3.70	0.65	0.24	0.11	-	1.1
$k_2 = 1.11\times10^8$ s^{-1}	3.0	10.3	1.78	3.12	0.58	0.40	0.02	-	1.1
τ_M = 270 ns	3.4	10.6	2.08	3.39	0.51	0.48	0.02	-	1.1
	3.8	10.0	2.54	2.29	0.46	0.53	0.01	-	1.0
	1.2	10.5	0.89	3.30	0.61	0.20	0.18	-	1.2
PyEG$_5$-PEG$_4$MA	1.9	11.0	1.08	3.00	0.64	0.24	0.12	-	1.1
$k_2 = 0.91\times10^8$ s^{-1}	2.2	9.2	1.30	2.59	0.61	0.30	0.09	-	1.0
τ_M = 270 ns	2.4	8.9	1.39	2.75	0.59	0.33	0.07	-	1.2
	3.1	9.0	1.76	2.78	0.57	0.40	0.04	-	1.0
	2.2	13.4	1.15	4.60	0.65	0.23	0.13	-	1.1
PyEG$_5$-PEG$_5$MA	3.6	12.2	1.65	3.19	0.63	0.34	0.03	-	1.2
$k_2 = 1.43\times10^8$ s^{-1}	3.8	13.1	1.62	3.64	0.61	0.36	0.03	-	1.1
τ_M = 270 ns	4.8	11.6	2.13	3.21	0.56	0.43	0.02	-	1.2
	6.5	11.9	2.47	2.94	0.49	0.50	0.01	-	1.1
PyEG$_5$-PEG$_7$MA	2.2	18.1	1.10	3.82	0.43	0.16	0.09	0.33	1.2
$k_2 = 1.49\times10^8$ s^{-1}	3.0	17.7	1.26	3.06	0.42	0.21	0.04	0.33	1.1
τ_M = 270 ns	3.6	15.1	1.42	2.97	0.44	0.23	0.04	0.30	1.2
τ_S = 4 ns	4.2	17.0	1.59	2.76	0.43	0.28	0.03	0.26	1.2
	2.9	12.3	1.31	2.88	0.64	0.21	0.15	-	1.1
PyEG$_5$-PEG$_9$MA	5.2	13.8	1.76	3.23	0.61	0.35	0.04	-	1.1
$k_2 = 1.40\times10^8$ s^{-1}	5.8	14.2	1.82	3.16	0.55	0.41	0.04	-	1.2
τ_M = 270 ns	7.4	13.0	2.30	2.68	0.51	0.48	0.02	-	1.1
	10.2	12.2	3.12	1.98	0.36	0.63	0.01	-	1.1
PyEG$_5$-	2.1	13.8	1.24	2.97	0.50	0.15	0.35	-	1.1
PEG$_{19}$MA	3.2	12.7	1.39	2.97	0.59	0.22	0.19	-	1.2
$k_2 = 1.33\times10^8$ s^{-1}	5.2	13.9	1.76	2.71	0.53	0.34	0.13	-	1.1
τ_M = 270 ns	6.2	12.4	1.93	2.51	0.57	0.38	0.06	-	1.1
	6.6	14.6	2.01	2.26	0.51	0.42	0.07	-	1.1

Table S2. Parameters retrieved from the FBM analysis of the monomer decays for the $PyEG_5$-PEG_nMA samples in degassed THF.

Sample	Mol%	k_{blob} ($10^6 s^{-1}$)	<n>	$k_e[blob]$ ($10^6 s^{-1}$)	f_{Mdiff}	f_{k2}	f_{Mfree}	χ^2
$PyEG_5$-PEG_0MA $k_2 = 1.02\times10^8$ s^{-1} τ_M = 270 ns	0.9	9.1	1.01	3.40	0.71	0.14	0.15	1.2
	2.0	9.5	1.74	3.84	0.69	0.27	0.04	1.1
	2.6	9.8	2.15	4.33	0.63	0.35	0.02	1.1
	3.3	9.1	2.79	4.10	0.57	0.43	0.00	1.1
	3.3	7.9	2.86	2.04	0.58	0.41	0.00	1.2
$PyEG_5$-PEG_3MA $k_2 = 0.71\times10^8$ s^{-1} τ_M = 270 ns	2.0	7.8	1.17	3.88	0.64	0.26	0.09	1.1
	2.1	7.5	1.00	3.47	0.66	0.25	0.09	1.1
	3.0	6.8	1.66	3.06	0.61	0.35	0.03	1.1
	3.4	6.5	2.01	2.74	0.56	0.43	0.01	1.1
	3.8	6.2	2.47	2.89	0.51	0.48	0.01	1.1
$PyEG_5$-PEG_4MA $k_2 = 0.68\times10^8$ s^{-1} τ_M = 270 ns	1.2	9.6	0.76	2.94	0.63	0.16	0.21	1.2
	1.9	8.1	0.99	2.69	0.65	0.21	0.21	1.1
	2.2	6.7	1.22	2.66	0.65	0.23	0.12	1.1
	2.4	6.5	1.33	2.48	0.65	0.26	0.09	1.0
	3.1	6.2	1.65	2.28	0.60	0.36	0.04	1.1
$PyEG_5$-PEG_5MA $k_2 = 0.88\times10^8$ s^{-1} τ_M = 270 ns	2.2	9.2	1.05	3.80	0.65	0.22	0.13	1.2
	3.6	8.3	1.57	3.26	0.62	0.33	0.05	1.1
	3.8	8.7	1.53	3.22	0.63	0.33	0.04	1.1
	4.8	7.9	2.01	3.25	0.57	0.41	0.02	1.1
	6.5	7.7	2.43	2.81	0.49	0.50	0.01	1.0
$PyEG_5$-PEG_7MA $k_2 = 0.80\times10^8$ s^{-1} τ_M = 270 ns	2.2	9.4	1.02	3.99	0.58	0.23	0.20	1.1
	3.0	9.9	1.13	3.55	0.61	0.29	0.10	1.1
	3.6	8.9	1.27	3.57	0.62	0.30	0.08	1.1
	4.2	9.7	1.36	3.50	0.57	0.37	0.06	1.1
$PyEG_5$-PEG_9MA $k_2 = 0.91\times10^8$ s^{-1} τ_M = 270 ns	2.9	10.1	1.12	3.64	0.62	0.22	0.16	1.1
	5.2	8.9	1.67	2.90	0.61	0.36	0.04	1.1
	5.8	9.0	1.83	2.81	0.57	0.40	0.03	1.1
	7.4	8.6	2.21	2.78	0.53	0.46	0.02	1.1
	10.2	7.4	3.31	2.86	0.40	0.60	0.00	1.1
$PyEG_5$-$PEG_{19}MA$ $k_2 = 0.86\times10^8$ s^{-1} τ_M = 270 ns	2.1	10.7	1.03	4.73	0.48	0.15	0.37	1.2
	3.2	11.3	1.13	3.84	0.59	0.24	0.17	1.2
	4.4	8.8	1.44	3.96	0.60	0.23	0.16	1.1
	5.2	10.4	1.53	3.42	0.53	0.33	0.13	1.2
	6.2	8.3	1.83	2.48	0.60	0.35	0.05	1.2
	6.6	10.0	1.77	2.69	0.53	0.41	0.06	1.2

Table S3. Parameters retrieved from the FBM analysis of the monomer decays for the $PyEG_5$-PEG_nMA samples in degassed DMF.

Sample	Mol%	k_{blob} ($10^6 s^{-1}$)	<n>	$k_e[blob]$ ($10^6 s^{-1}$)	f_{Mdiff}	f_{k2}	f_{Mfree}	χ^2
$PyEG_5$-PEG_0MA $k_2 = 0.80 \times 10^8$ s^{-1} τ_M = 230 ns	0.9	9.3	0.87	4.64	0.69	0.08	0.17	1.1
	2.0	8.3	1.59	3.99	0.70	0.08	0.04	1.1
	2.6	7.2	2.14	3.13	0.65	0.08	0.02	1.1
	3.3	6.9	2.76	2.20	0.59	0.08	0.00	1.1
	3.3	7.1	2.59	2.67	0.60	0.08	0.01	1.1
$PyEG_5$-PEG_3MA $k_2 = 0.73 \times 10^8$ s^{-1} τ_M = 230 ns	2.0	6.9	1.15	3.34	0.68	0.19	0.13	1.2
	2.1	7.7	0.91	4.16	0.60	0.20	0.20	1.2
	3.0	6.7	1.48	3.67	0.68	0.26	0.06	1.1
	3.4	6.8	1.74	3.03	0.65	0.31	0.03	1.1
	3.8	6.2	2.10	2.83	0.62	0.37	0.01	1.1
$PyEG_5$-PEG_4MA $k_2 = 0.62 \times 10^8$ s^{-1} τ_M = 230 ns	1.2	7.3	0.81	3.24	0.60	0.14	0.26	1.1
	1.9	7.9	0.87	3.75	0.62	0.18	0.20	1.1
	2.2	6.3	1.10	2.52	0.68	0.17	0.14	1.1
	2.4	6.2	1.18	2.67	0.67	0.21	0.12	1.1
	3.1	6.1	1.46	2.53	0.66	0.28	0.06	1.1
$PyEG_5$-PEG_5MA $k_2 = 0.69 \times 10^8$ s^{-1} τ_M = 230 ns	2.2	7.2	1.09	3.38	0.64	0.21	0.15	1.0
	3.6	6.8	1.48	2.78	0.63	0.31	0.06	1.0
	3.8	6.9	1.51	3.32	0.63	0.31	0.05	1.0
	4.8	6.3	1.96	2.51	0.58	0.39	0.03	1.1
	6.5	5.3	2.53	1.43	0.51	0.48	0.01	1.2
$PyEG_5$-PEG_7MA $k_2 = 0.58 \times 10^8$ s^{-1} τ_M = 230 ns	2.2	10.3	0.87	4.56	0.58	0.20	0.21	1.1
	3.0	8.8	0.97	4.84	0.55	0.30	0.15	1.1
	3.6	7.6	1.14	3.37	0.60	0.31	0.09	1.2
	4.2	8.1	1.22	3.87	0.54	0.39	0.08	1.1
$PyEG_5$-PEG_9MA $k_2 = 1.03 \times 10^8$ s^{-1} τ_M = 230 ns	2.9	10.30	0.97	4.76	0.68	0.14	0.18	1.0
	5.2	10.12	1.34	3.91	0.71	0.23	0.06	1.1
	5.8	11.52	1.37	3.96	0.69	0.25	0.06	1.1
	7.4	10.57	1.71	3.81	0.64	0.29	0.07	1.1
	10.2	9.22	2.55	3.27	0.56	0.43	0.01	1.2
$PyEG_5$-$PEG_{19}MA$ $k_2 = 0.83 \times 10^8$ s^{-1} τ_M = 230 ns	2.1	11.6	0.87	4.64	0.47	0.12	0.40	1.1
	3.2	10.0	1.05	4.14	0.60	0.18	0.23	1.1
	4.4	11.5	1.15	3.72	0.61	0.23	0.16	1.2
	5.2	10.4	1.35	3.70	0.57	0.26	0.16	1.1
	6.2	8.0	1.70	3.17	0.64	0.28	0.07	1.0
	6.6	11.5	1.45	3.06	0.57	0.36	0.07	1.2

Table S4. Parameters retrieved from the FBM analysis of the monomer decays for the PyEG$_5$-PEG$_n$MA samples in aerated DMSO.

Sample	Mol%	k_{blob} ($10^6 s^{-1}$)	<n>	$k_e[blob]$ ($10^6 s^{-1}$)	f_{Mdiff}	f_{k2}	f_{Mfree}	f_{MS}	χ^2
PyEG$_5$-PEG$_0$MA $k_2 = 1.43\times10^8$ s^{-1} τ_M = 120 ns	0.9	9.4	0.81	6.91	0.74	0.07	0.19	-	1.1
	2.0	8.6	1.38	5.73	0.82	0.13	0.05	-	1.1
	2.6	9.2	1.63	6.18	0.80	0.16	0.03	-	1.2
	3.3	8.2	2.08	4.68	0.79	0.20	0.01	-	1.1
	3.3	10.0	1.74	4.93	0.76	0.21	0.02	-	1.2
PyEG$_5$-PEG$_3$MA $k_2 = 0.87\times10^8$ s^{-1} τ_M = 120 ns	1.6	9.7	0.69	8.16	0.69	0.08	0.25	-	1.2
	2.0	7.1	1.06	6.01	0.77	0.14	0.09	-	1.1
	3.0	7.5	1.30	6.15	0.76	0.17	0.06	-	1.1
	3.4	7.9	1.49	6.51	0.74	0.21	0.05	-	1.1
	3.8	7.1	1.81	5.33	0.73	0.24	0.03	-	1.0
PyEG$_5$-PEG$_4$MA $k_2 = 0.83\times10^8$ s^{-1} τ_M = 120 ns	1.2	8.1	0.73	7.07	0.69	0.08	0.22	-	1.1
	1.9	8.1	0.84	6.64	0.70	0.10	0.20	-	1.1
	2.2	7.5	0.98	7.22	0.70	0.10	0.20	-	1.1
	2.4	6.9	1.06	5.47	0.75	0.13	0.12	-	1.2
	3.1	8.4	1.17	7.88	0.74	0.16	0.10	-	1.1
PyEG$_5$-PEG$_5$MA $k_2 = 0.76\times10^8$ s^{-1} τ_M = 120 ns	2.2	8.3	0.85	5.62	0.68	0.13	0.19	-	1.1
	3.6	8. 9	1.01	5.18	0.73	0.18	0.09	-	1.1
	3.8	8.0	1.13	4.89	0.73	0.20	0.07	-	1.1
	4.8	7.8	1.38	4.16	0.74	0.24	0.02	-	1.1
	6.5	7.3	1.72	4.62	0.68	0.29	0.03	-	1.2
PyEG$_5$-PEG$_7$MA $k_2 = 0.83\times10^8$ s^{-1} τ_M = 120 ns τ_S = 4 ns	2.2	13.6	0.72	5.48	0.35	0.09	0.08	0.48	1.0
	3.0	12.9	0.77	5.22	0.38	0.11	0.06	0.46	1.0
	3.6	9.9	1.00	5.90	0.49	0.13	0.07	0.31	1.1
	4.2	12.1	0.96	5.88	0.41	0.13	0.04	0.41	1.1
PyEG$_5$-PEG$_9$MA $k_2 = 1.05\times10^8$ s^{-1} τ_M = 120 ns	2.9	11.1	0.78	6.53	0.67	0.10	0.23	-	1.1
	5.2	9.4	1.20	5.20	0.75	0.17	0.08	-	1.1
	5.8	9.7	1.26	5.25	0.75	0.18	0.07	-	1.1
	7.4	9.3	1.53	4.98	0.75	0.22	0.02	-	1.1
	10.2	9.2	2.02	3.93	0.69	0.31	0.00	-	1.2
PyEG$_5$-PEG$_{19}$MA $k_2 = 1.09\times10^8$ s^{-1} τ_M = 120 ns	2.1	17.5	0.54	3.71	0.45	0.07	0.48	-	1.0
	3.2	15.5	0.65	4.11	0.55	0.09	0.36	-	1.1
	5.2	12.6	0.99	4.48	0.67	0.15	0.18	-	1.0
	6.2	9.0	1.18	4.74	0.71	0.14	0.14	-	1.2
	6.6	13.5	0.95	3.62	0.78	0.20	0.03	-	1.2

Table S5. Parameters retrieved from the FBM analysis of the excimer decays for the PyEG5-PEGnMA samples in degassed acetonitrile.

Sample	Mol %	f_{Ek2}	τ_{E0} (ns)	$f_{EdiffE0}$	f_{EE0}	τ_D (ns)	f_{EdiffD}	f_{ED}	f_{ES}	χ^2
PyEG5-PEG0MA	0.9	0.25	49	0.47	0.00	59	0.24	0.03	-	1.0
	2.0	0.39	47	0.23	0.00	55	0.31	0.08	-	1.2
	2.6	0.49	47	0.21	0.00	52	0.22	0.08	-	1.1
	3.3	0.55	46	0.15	0.00	54	0.18	0.12	-	1.2
	3.3	0.55	46	0.18	0.01	54	0.17	0.09	-	1.1
PyEG5-PEG3MA	2.0	0.29	34	0.00	0.07	53	0.64	0.00	-	1.0
	2.1	0.31	47	0.29	0.17	66	0.31	0.01	-	1.1
	3.0	0.38	46	0.25	0.00	54	0.31	0.06	-	1.1
	3.4	0.44	50	0.39	0.00	54	0.08	0.08	-	1.1
	3.8	0.48	46	0.29	0.00	55	0.13	0.11	-	1.0
PyEG5-PEG4MA	1.2	0.24	27	0.04	0.03	55	0.69	0.00	-	1.2
	1.9	0.27	27	0.14	0.03	56	0.56	0.00	-	1.1
	2.2	0.32	25	0.01	0.04	61	0.64	0.00		1.0
	2.4	0.34	48	0.35	0.05	56	0.26	0.00	-	1.2
	3.1	0.39	46	0.49	0.01	70	0.06	0.05	-	1.0
PyEG5-PEG5MA	2.2	0.25	53	0.72	0.03	-	-	-	-	1.1
	3.2	0.56	53	0.61	0.05	-	-	-	-	1.2
	3.6	0.35	54	0.60	0.05	-	-	-	-	1.1
	4.8	0.41	52	0.53	0.06	-	-	-	-	1.2
	6.5	0.46	52	0.46	0.08	-	-	-	-	1.1
PyEG5-PEG7MA τ_S = 4 ns	2.2	0.25	26	0.02	0.05	59	0.68	0.00	0.00	1.2
	3.0	0.32	49	0.29	0.04	68	0.34	0.00	0.01	1.1
	3.6	0.32	43	0.27	0.02	63	0.36	0.00	0.03	1.2
	4.2	0.36	48	0.33	0.05	67	0.23	0.00	0.02	1.2
PyEG5-PEG9MA	2.9	0.24	42	0.48	0.00	66	0.25	0.03	-	1.1
	5.2	0.35	44	0.24	0.00	55	0.36	0.06	-	1.1
	5.8	0.40	49	0.33	0.00	55	0.21	0.05		1.2
	7.4	0.45	45	0.26	0.00	55	0.21	0.08	-	1.1
	10.2	0.57	45	0.15	0.10	54	0.18	0.00	-	1.1
PyEG5-PEG19MA	2.1	0.22	44	0.42	0.00	67	0.32	0.03	-	1.1
	3.2	0.27	47	0.51	0.00	64	0.19	0.04	-	1.2
	5.2	0.37	41	0.11	0.00	54	0.47	0.05	-	1.1
	6.2	0.38	43	0.22	0.00	54	0.35	0.05	-	1.1
	6.6	0.42	46	0.33	0.03	62	0.18	0.04	-	1.1

Table S6. Parameters retrieved from the FBM analysis of the excimer decays for the $PyEG_5$-PEG_nMA samples in degassed THF.

Sample	Mol %	f_{Ek2}	τ_{E0} (ns)	$f_{EdiffE0}$	f_{EE0}	τ_D (ns)	f_{EdiffD}	f_{ED}	χ^2
$PyEG_5$-PEG_0MA	0.9	0.17	56	0.82	0.01	-	-	-	1.2
	2.0	0.28	53	0.71	0.01	-	-	-	1.1
	2.6	0.35	52	0.63	0.02	-	-	-	1.1
	3.3	0.42	51	0.56	0.02	-	-	-	1.1
	3.3	0.41	51	0.57	0.02	-	-	-	1.2
$PyEG_5$-PEG_3MA	2.0	0.27	52	0.53	0.04	70	0.13	0.03	1.1
	2.1	0.23	55	0.52	0.17	83	0.07	0.02	1.1
	3.0	0.34	48	0.32	0.00	59	0.28	0.06	1.1
	3.4	0.41	52	0.33	0.00	55	0.20	0.06	1.1
	3.8	0.45	49	0.33	0.00	57	0.14	0.09	1.1
$PyEG_5$-PEG_4MA	1.2	0.20	42	0.41	0.04	73	0.35	0.00	1.2
	1.9	0.24	51	0.61	0.04	90	0.11	0.00	1.1
	2.2	0.26	42	0.47	0.01	65	0.26	0.01	1.1
	2.4	0.27	44	0.45	0.05	66	0.22	0.01	1.0
	3.1	0.37	46	0.37	0.01	60	0.25	0.01	1.1
$PyEG_5$-PEG_5MA	2.2	0.25	47	0.42	0.00	64	0.32	0.02	1.2
	3.2	0.34	49	0.51	0.00	68	0.12	0.03	1.1
	3.6	0.34	47	0.42	0.00	62	0.22	0.03	1.1
	4.8	0.41	34	0.02	0.03	52	0.54	0.00	1.1
	6.5	0.48	50	0.32	0.05	54	0.16	0.00	1.0
$PyEG_5$-PEG_7MA	2.2	0.27	49	0.47	0.02	67	0.23	0.02	1.1
	3.0	0.31	51	0.53	0.00	78	0.13	0.00	1.1
	3.6	0.32	51	0.58	0.01	74	0.07	0.01	1.1
	4.2	0.38	54	0.54	0.02	80	0.04	0.02	1.1
$PyEG_5$-PEG_9MA	2.9	0.25	51	0.57	0.00	75	0.15	0.03	1.1
	5.2	0.36	49	0.42	0.00	61	0.20	0.03	1.1
	5.8	0.41	50	0.50	0.00	69	0.08	0.01	1.1
	7.4	0.44	51	0.50	0.00	70	0.01	0.05	1.1
	10.2	0.55	48	0.27	0.00	55	0.10	0.08	1.1
$PyEG_5$-$PEG_{19}MA$	2.1	0.23	58	0.75	0.02	-	-	-	1.2
	3.2	0.27	59	0.69	0.04	-	-	-	1.2
	4.4	0.27	54	0.69	0.05	-	-	-	1.1
	5.2	0.37	56	0.60	0.03	-	-	-	1.2
	6.2	0.35	52	0.60	0.05	-	-	-	1.2
	6.6	0.42	54	0.53	0.05	-	-	-	1.2

Table S7. Parameters retrieved from the FBM analysis of the excimer decays for the $PyEG_5$-PEG_nMA samples in degassed DMF.

Sample	Mol %	f_{Ek2}	τ_{E0} (ns)	$f_{EdiffE0}$	f_{EE0}	τ_D (ns)	f_{EdiffD}	f_{ED}	χ^2
$PyEG_5$-PEG_0MA	0.9	0.17	55	0.81	0.02	-	-	-	1.1
	2.0	0.27	50	0.71	0.02	-	-	-	1.1
	2.6	0.32	48	0.65	0.03	-	-	-	1.1
	3.3	0.40	49	0.57	0.03	-	-	-	1.1
	3.3	0.39	49	0.59	0.03	-	-	-	1.1
$PyEG_5$-PEG_3MA	2.0	0.20	46	0.64	0.02	83	0.10	0.04	1.2
	2.1	0.20	55	0.49	0.20	99	0.08	0.03	1.2
	3.0	0.26	49	0.64	0.04	73	0.05	0.01	1.1
	3.4	0.31	46	0.45	0.04	61	0.20	0.00	1.1
	3.8	0.36	48	0.41	0.02	52	0.21	0.00	1.1
$PyEG_5$-PEG_4MA	1.2	0.19	44	0.51	0.01	70	0.28	0.00	1.1
	1.9	0.23	48	0.52	0.02	69	0.24	0.00	1.1
	2.2	0.20	38	0.55	0.01	65	0.24	0.01	1.1
	2.4	0.23	41	0.48	0.04	62	0.25	0.00	1.1
	3.1	0.29	42	0.44	0.02	60	0.25	0.00	1.1
$PyEG_5$-PEG_5MA	2.2	0.24	45	0.51	0.00	62	0.23	0.02	1.0
	3.2	0.32	50	0.65	0.02	128	0.00	0.01	1.0
	3.6	0.32	47	0.47	0.03	57	0.18	0.00	1.0
	4.8	0.39	45	0.45	0.00	61	0.13	0.03	1.1
	6.5	0.47	49	0.30	0.01	49	0.20	0.02	1.2
$PyEG_5$-PEG_7MA	2.2	0.25	18	0.20	0.02	57	0.53	0.00	1.1
	3.0	0.34	54	0.43	0.04	64	0.20	0.00	1.1
	3.6	0.33	49	0.46	0.04	66	0.18	0.00	1.2
	4.2	0.40	51	0.31	0.04	61	0.25	0.00	1.1
$PyEG_5$-PEG_9MA	2.9	0.17	53	0.82	0.01	-	-	-	1.0
	5.2	0.24	53	0.75	0.02	-	-	-	1.1
	5.8	0.26	54	0.72	0.02	-	-	-	1.1
	7.4	0.30	52	0.68	0.02	-	-	-	1.1
	10.2	0.42	51	0.55	0.02	-	-	-	1.2
$PyEG_5$-$PEG_{19}MA$	2.1	0.20	61	0.78	0.02	-	-	-	1.1
	3.2	0.22	56	0.76	0.02	-	-	-	1.1
	4.4	0.27	61	0.71	0.02	-	-	-	1.2
	5.2	0.31	54	0.67	0.03	-	-	-	1.1
	6.2	0.30	49	0.68	0.02	-	-	-	1.0
	6.6	0.37	55	0.60	0.03	-	-	-	1.2

Table S8. Parameters retrieved from the FBM analysis of the excimer decays for the $PyEG_5$-PEG_nMA samples in aerated DMSO.

Sample	Mol %	f_{Ek2}	τ_{E0} (ns)	$f_{EdiffE0}$	f_{EE0}	τ_D (ns)	f_{EdiffD}	f_{ED}	f_{ES}	χ^2
$PyEG_5$-PEG_0MA	0.9	0.08	40	0.90	0.02	-	-	-	-	1.1
	2.0	0.14	39	0.84	0.02	-	-	-	-	1.1
	2.6	0.17	38	0.81	0.03	-	-	-	-	1.2
	3.3	0.19	39	0.78	0.03	-	-	-	-	1.1
	3.3	0.21	41	0.76	0.03	-	-	-	-	1.2
$PyEG_5$-PEG_3MA	1.6	0.10	41	0.81	0.09	-	-	-	-	1.2
	2.0	0.14	39	0.80	0.05	-	-	-	-	1.1
	3.0	0.18	39	0.78	0.05	-	-	-	-	1.1
	3.4	0.21	39	0.75	0.04	-	-	-	-	1.1
	3.8	0.24	38	0.73	0.03	-	-	-	-	1.0
$PyEG_5$-PEG_4MA	1.2	0.10	40	0.88	0.02	-	-	-	-	1.1
	1.9	0.13	40	0.85	0.03	-	-	-	-	1.1
	2.2	0.13	38	0.85	0.02	-	-	-	-	1.1
	2.4	0.14	39	0.81	0.05	-	-	-	-	1.2
	3.1	0.17	39	0.81	0.02	-	-	-	-	1.1
$PyEG_5$-PEG_5MA	2.2	0.15	43	0.82	0.02	-	-	-	-	1.1
	3.2	0.19	44	0.77	0.03	-	-	-	-	1.1
	3.6	0.21	43	0.76	0.03				-	1.1
	4.8	0.24	42	0.73	0.03	-	-	-	-	1.1
	6.5	0.29	42	0.67	0.03	-	-	-	-	1.2
$PyEG_5$-PEG_7MA τ_S = 4 ns	2.2	0.19	49	0.74	0.04	-	-	-	0.03	1.0
	3.0	0.21	48	0.72	0.03	-	-	-	0.04	1.0
	3.6	0.21	43	0.75	0.03	-	-	-	0.01	1.1
	4.2	0.23	45	0.71	0.04	-	-	-	0.02	1.1
$PyEG_5$-PEG_9MA	2.9	0.12	44	0.85	0.02	-	-	-	-	1.1
	5.2	0.18	42	0.80	0.02	-	-	-	-	1.1
	5.8	0.19	42	0.79	0.02	-	-	-	-	1.1
	7.4	0.22	41	0.75	0.03	-	-	-	-	1.1
	10.2	0.30	41	0.67	0.04	-	-	-	-	1.2
$PyEG_5$-$PEG_{19}MA$	2.1	0.12	60	0.85	0.03	-	-	-	-	1.1
	3.2	0.13	57	0.83	0.03	-	-	-	-	1.2
	5.2	0.18	49	0.79	0.03	-	-	-	-	1.0
	6.2	0.17	44	0.81	0.02	-	-	-	-	1.1
	6.6	0.19	48	0.77	0.03	-	-	-	-	1.2

Table S9. Molar fractions retrieved from the FBM analysis of the monomer and excimer decays for the PyEG5-PEGnMA samples in degassed acetonitrile.

Sample	Mol %	f_{free}	f_{agg}	f_{diff}	f_{k2}	χ^2
PyEG5-PEG0MA	0.9	0.06	0.03	0.75	0.16	1.0
	2.0	0.01	0.10	0.69	0.19	1.2
	3.0	0.01	0.12	0.64	0.22	1.1
	3.4	0.00	0.20	0.55	0.25	1.2
	3.8	0.00	0.17	0.58	0.25	1.1
PyEG5-PEG3MA	2.0	0.05	0.06	0.61	0.28	1.0
	2.1	0.06	0.15	0.51	0.27	1.1
	3.0	0.01	0.06	0.55	0.38	1.1
	3.4	0.01	0.08	0.47	0.44	1.1
	3.8	0.01	0.11	0.41	0.47	1.0
PyEG5-PEG4MA	1.2	0.14	0.02	0.63	0.21	1.2
	1.9	0.09	0.03	0.64	0.24	1.1
	2.2	0.06	0.03	0.61	0.30	1.0
	2.4	0.05	0.05	0.58	0.33	1.2
	3.1	0.02	0.06	0.54	0.38	1.0
PyEG5-PEG5MA	2.2	0.09	0.03	0.65	0.23	1.1
	3.2	0.02	0.04	0.50	0.45	1.2
	3.6	0.02	0.05	0.59	0.34	1.1
	4.8	0.01	0.06	0.53	0.40	1.2
	6.5	0.01	0.08	0.66	0.29	1.1
PyEG5-PEG7MA	2.2	0.06	0.05	0.65	0.24	1.2
	3.0	0.03	0.04	0.62	0.31	1.1
	3.6	0.02	0.02	0.63	0.33	1.2
	4.2	0.02	0.05	0.57	0.37	1.2
PyEG5-PEG9MA	2.9	0.11	0.03	0.65	0.21	1.1
	5.2	0.02	0.06	0.58	0.34	1.1
	5.8	0.02	0.05	0.53	0.40	1.2
	7.4	0.01	0.07	0.47	0.44	1.1
	10.2	0.00	0.10	0.33	0.57	1.1
PyEG5-PEG19MA	2.1	0.28	0.02	0.53	0.16	1.1
	3.2	0.14	0.03	0.60	0.23	1.2
	5.2	0.08	0.05	0.53	0.34	1.1
	6.2	0.03	0.05	0.55	0.37	1.1
	6.6	0.04	0.06	0.49	0.41	1.1

Table S10. Molar fractions retrieved from the FBM analysis of the monomer and excimer decays for the $PyEG_5$-PEG_nMA samples in degassed THF.

Sample	Mol %	f_{free}	f_{agg}	f_{diff}	f_{k2}	χ^2
$PyEG_5$-PEG_0MA	0.9	0.14	0.01	0.76	0.15	1.2
	2.0	0.03	0.01	0.84	0.33	1.1
	2.6	0.02	0.03	0.82	0.45	1.1
	3.3	0.00	0.02	0.82	0.62	1.1
	3.3	0.00	0.03	0.82	0.58	1.2
$PyEG_5$-PEG_3MA	2.0	0.06	0.07	0.62	0.25	1.1
	2.1	0.05	0.17	0.56	0.21	1.1
	3.0	0.02	0.06	0.58	0.34	1.1
	3.4	0.01	0.06	0.53	0.41	1.1
	3.8	0.00	0.08	0.47	0.44	1.1
$PyEG_5$-PEG_4MA	1.2	0.17	0.03	0.64	0.16	1.2
	1.9	0.16	0.03	0.60	0.20	1.1
	2.2	0.09	0.02	0.66	0.23	1.1
	2.4	0.06	0.05	0.63	0.26	1.0
	3.1	0.02	0.02	0.60	0.36	1.1
$PyEG_5$-PEG_5MA	2.2	0.10	0.02	0.66	0.22	1.2
	3.2	0.03	0.03	0.61	0.33	1.1
	3.6	0.03	0.03	0.62	0.33	1.1
	4.8	0.01	0.03	0.55	0.41	1.1
	6.5	0.00	0.05	0.47	0.48	1.0
$PyEG_5$-PEG_7MA	2.2	0.15	0.02	0.60	0.23	1.1
	3.0	0.07	0.03	0.61	0.29	1.1
	3.6	0.05	0.03	0.62	0.30	1.1
	4.2	0.04	0.04	0.56	0.37	1.1
$PyEG_5$-PEG_9MA	2.9	0.12	0.03	0.63	0.22	1.1
	5.2	0.02	0.03	0.60	0.35	1.1
	5.8	0.02	0.01	0.57	0.40	1.1
	7.4	0.01	0.05	0.51	0.44	1.1
	10.2	0.00	0.08	0.37	0.55	1.1
$PyEG_5$-$PEG_{19}MA$	2.1	0.30	0.01	0.52	0.16	1.2
	3.2	0.13	0.03	0.60	0.24	1.2
	4.4	0.12	0.04	0.61	0.23	1.1
	5.2	0.08	0.03	0.54	0.34	1.2
	6.2	0.03	0.05	0.58	0.34	1.2
	6.6	0.03	0.04	0.52	0.41	1.2

Table S11. Molar fractions retrieved from the FBM analysis of the monomer and excimer decays for the $PyEG_5$-PEG_nMA samples in degassed DMF.

Sample	Mol %	f_{free}	f_{agg}	f_{diff}	f_{k2}	
$PyEG_5$-PEG_0MA	0.9	0.16	0.02	0.75	0.16	1.1
	2.0	0.03	0.02	0.85	0.32	1.1
	2.6	0.02	0.03	0.84	0.42	1.1
	3.3	0.00	0.04	0.84	0.59	1.1
	3.3	0.00	0.04	0.84	0.56	1.1
$PyEG_5$-PEG_3MA	2.0	0.10	0.05	0.66	0.18	1.2
	2.1	0.12	0.20	0.50	0.17	1.2
	3.0	0.04	0.05	0.66	0.25	1.1
	3.4	0.02	0.04	0.64	0.31	1.1
	3.8	0.01	0.02	0.61	0.36	1.1
$PyEG_5$-PEG_4MA	1.2	0.21	0.01	0.62	0.15	1.1
	1.9	0.16	0.02	0.64	0.19	1.1
	2.2	0.12	0.01	0.70	0.18	1.1
	2.4	0.09	0.04	0.66	0.20	1.1
	3.1	0.04	0.02	0.66	0.28	1.1
$PyEG_5$-PEG_5MA	2.2	0.11	0.02	0.66	0.21	1.0
	3.2	0.04	0.02	0.63	0.31	1.0
	3.6	0.04	0.03	0.63	0.31	1.0
	4.8	0.02	0.03	0.57	0.38	1.1
	6.5	0.00	0.03	0.50	0.47	1.2
$PyEG_5$-PEG_7MA	2.2	0.17	0.02	0.61	0.21	1.1
	3.0	0.10	0.03	0.56	0.30	1.1
	3.6	0.06	0.03	0.60	0.31	1.2
	4.2	0.04	0.04	0.53	0.38	1.1
$PyEG_5$-PEG_9MA	2.9	0.16	0.01	0.69	0.14	1.0
	5.2	0.05	0.01	0.71	0.23	1.1
	5.8	0.05	0.02	0.68	0.25	1.1
	7.4	0.05	0.02	0.64	0.29	1.1
	10.2	0.01	0.02	0.55	0.42	1.2
$PyEG_5$-$PEG_{19}MA$	2.1	0.35	0.02	0.51	0.13	1.1
	3.2	0.18	0.02	0.62	0.18	1.1
	4.4	0.12	0.02	0.62	0.24	1.2
	5.2	0.11	0.02	0.59	0.27	1.1
	6.2	0.05	0.02	0.64	0.28	1.0
	6.6	0.04	0.03	0.57	0.36	1.2

Table S12. Molar fractions retrieved from the FBM analysis of the monomer and excimer decays for the $PyEG_5$-PEG_nMA samples in aerated DMSO.

Sample	Mol %	f_{free}	f_{agg}	f_{diff}	f_{k2}	χ^2
$PyEG_5$-PEG_0MA	0.9	0.17	0.02	0.70	0.11	1.1
	2.0	0.04	0.02	0.80	0.14	1.1
	2.6	0.03	0.03	0.80	0.14	1.2
	3.3	0.01	0.03	0.81	0.15	1.1
	3.3	0.02	0.03	0.80	0.15	1.2
$PyEG_5$-PEG_3MA	1.6	0.22	0.07	0.63	0.08	1.2
	2.0	0.07	0.05	0.74	0.13	1.1
	3.0	0.05	0.04	0.74	0.17	1.1
	3.4	0.04	0.04	0.72	0.21	1.1
	3.8	0.02	0.03	0.71	0.23	1.0
$PyEG_5$-PEG_4MA	1.2	0.20	0.02	0.70	0.08	1.1
	1.9	0.17	0.02	0.70	0.10	1.1
	2.2	0.17	0.02	0.70	0.10	1.1
	2.4	0.10	0.04	0.73	0.13	1.2
	3.1	0.09	0.02	0.74	0.16	1.1
$PyEG_5$-PEG_5MA	2.2	0.16	0.02	0.69	0.13	1.1
	3.2	0.07	0.03	0.72	0.18	1.1
	3.6	0.05	0.03	0.72	0.20	1.1
	4.8	0.02	0.03	0.72	0.23	1.1
	6.5	0.02	0.03	0.66	0.29	1.2
$PyEG_5$-PEG_7MA	2.2	0.04	0.03	0.71	0.18	1.0
	3.0	0.04	0.03	0.72	0.20	1.0
	3.6	0.04	0.02	0.72	0.20	1.1
	4.2	0.03	0.04	0.70	0.23	1.1
$PyEG_5$-PEG_9MA	2.9	0.20	0.02	0.68	0.10	1.1
	5.2	0.07	0.02	0.75	0.16	1.1
	5.8	0.06	0.02	0.74	0.18	1.1
	7.4	0.02	0.03	0.74	0.22	1.1
	10.2	0.00	0.04	0.67	0.30	1.2
$PyEG_5$-$PEG_{19}MA$	2.1	0.44	0.02	0.47	0.07	1.1
	3.2	0.32	0.02	0.57	0.09	1.2
	5.2	0.15	0.03	0.67	0.15	1.0
	6.2	0.12	0.02	0.71	0.15	1.1
	6.6	0.02	0.03	0.76	0.19	1.2

G] References

1. Duhamel, J. New Insights in the Study of Pyrene Excimer Fluorescence to Characterize Macromolecules and their Supramolecular Assemblies in Solution. *Langmuir* **2012**, *28*, 6527-6538.
2. Duhamel, J. Global Analysis of Fluorescence Decays to Probe the Internal Dynamics of Fluorescently Labeled Macromolecules. *Langmuir* **2014**, *30*, 2307-2324.
